# Histone deacetylase 9 promotes endothelial-mesenchymal transition and an unfavorable atherosclerotic plaque phenotype

**DOI:** 10.1172/JCI131178

**Published:** 2021-08-02

**Authors:** Laura Lecce, Yang Xu, Bhargavi V’Gangula, Nirupama Chandel, Venu Pothula, Axelle Caudrillier, Maria Paola Santini, Valentina d’Escamard, Delaine K. Ceholski, Przemek A. Gorski, Lijiang Ma, Simon Koplev, Martin Mæng Bjørklund, Johan L.M. Björkegren, Manfred Boehm, Jacob Fog Bentzon, Valentin Fuster, Ha Won Kim, Neal L. Weintraub, Andrew H. Baker, Emily Bernstein, Jason C. Kovacic

**Affiliations:** 1 Cardiovascular Research Institute, Icahn School of Medicine at Mount Sinai, New York, New York, USA.; 2 Centre for Cardiovascular Science, University of Edinburgh, Edinburgh, United Kingdom.; 3 Department of Genetics and Genomic Sciences, Icahn School of Medicine at Mount Sinai, New York, New York, USA.; 4 Department of Clinical Medicine, Heart Diseases, Aarhus University, Aarhus, Denmark.; 5 Centro Nacional de Investigaciones Cardiovasculares (CNIC), Madrid, Spain.; 6 Integrated Cardio Metabolic Centre, Department of Medicine, Karolinska Institutet, Karolinska Universitetssjukhuset, Huddinge, Sweden.; 7 Laboratory of Cardiovascular Regenerative Medicine, Translational Vascular Medicine Branch, National Heart Lung and Blood Institute, NIH, Bethesda, Maryland, USA.; 8 Department of Medicine, Cardiology Division and Vascular Biology Center, Medical College of Georgia at Augusta University, Augusta, Georgia, USA.; 9 Departments of Oncological Sciences and Dermatology, Icahn School of Medicine at Mount Sinai, New York, New York, USA.; 10 Victor Chang Cardiac Research Institute, Darlinghurst, New South Wales, Australia.; 11 St. Vincent’s Clinical School, University of New South Wales, Sydney, Australia.

**Keywords:** Cardiology, Vascular Biology, Atherosclerosis, Endothelial cells, Epigenetics

## Abstract

Endothelial-mesenchymal transition (EndMT) is associated with various cardiovascular diseases and in particular with atherosclerosis and plaque instability. However, the molecular pathways that govern EndMT are poorly defined. Specifically, the role of epigenetic factors and histone deacetylases (HDACs) in controlling EndMT and the atherosclerotic plaque phenotype remains unclear. Here, we identified histone deacetylation, specifically that mediated by HDAC9 (a class IIa HDAC), as playing an important role in both EndMT and atherosclerosis. Using in vitro models, we found class IIa HDAC inhibition sustained the expression of endothelial proteins and mitigated the increase in mesenchymal proteins, effectively blocking EndMT. Similarly, ex vivo genetic knockout of *Hdac9* in endothelial cells prevented EndMT and preserved a more endothelial-like phenotype. In vivo, atherosclerosis-prone mice with endothelial-specific *Hdac9* knockout showed reduced EndMT and significantly reduced plaque area. Furthermore, these mice displayed a more favorable plaque phenotype, with reduced plaque lipid content and increased fibrous cap thickness. Together, these findings indicate that HDAC9 contributes to vascular pathology by promoting EndMT. Our study provides evidence for a pathological link among EndMT, HDAC9, and atherosclerosis and suggests that targeting of HDAC9 may be beneficial for plaque stabilization or slowing the progression of atherosclerotic disease.

## Introduction

Endothelial-mesenchymal transition (EndMT) is an essential process during cardiac development, contributing to cardiac valve formation and the stabilization of new vasculature in the developing embryo (1, 2). EndMT also plays a key role in adult tissue regeneration and wound healing, in which endothelial cells acquire fibroblast-like functions to participate in extracellular matrix remodeling and tissue rebuilding (1, 2). Under certain pathological conditions, EndMT contributes to the progression of chronic fibrosing states (3) and is reported to occur during pulmonary (4, 5), renal (6, 7), and cardiac fibrosis (8, 9). EndMT also participates in vascular remodeling and neointima formation, for example, as a response to vein graft transplantation into the arterial circulation (10). More recently, it was shown that EndMT is associated with atherosclerotic plaque burden (11) and an unstable plaque phenotype in both mice and humans (12, 13).

EndMT can be induced by a variety of stimuli, such as exposure to cytokines, growth factors, oxidative stress, and inflammatory signals. Several signaling pathways have been associated with EndMT, most notably the TGF-β pathway (2, 14). Conversely, the FGF pathway is known to inhibit EndMT by maintaining expression of EndMT-inhibitory miRNAs, which belong to the *let-7* miRNA family and the miRNA-17-92 cluster (15–17). Of interest, a recent study showed that the long noncoding antisense transcript of GATA6 regulates EndMT via downstream changes in histone methylation (18). Beyond this, however, little is known about the biologic pathways and signaling mechanisms governing EndMT and the higher-order regulators at the apex of these EndMT pathways (1).

Epigenetic mechanisms and regulators of posttranslational modifications (PTMs), such as acetylation and methylation, are known to govern epithelial-to-mesenchymal transition (EMT), which is closely related to EndMT (19–22). Acetylation is carefully controlled through a balance of histone deacetylases (HDACs) and histone acetyltransferases (HATs), which have antagonistic functions. HDACs are a family of enzymes categorized into 5 main groups, class I (HDAC1, -2, -3, -8), class IIa (HDAC4, -5, -7, -9), class IIb (HDAC6, -10), class III (Sirt1–7), and class IV (HDAC11), that are responsible for removing acetyl groups from histones and nonhistone proteins (23, 24). Aberrant HDAC expression and activity can promote EMT and cancer metastasis, while HDAC inhibitors can prevent EMT (25–27). Because EndMT is a specific form of EMT (1, 2), this provides a strong rationale for believing that HDACs may also be of importance in regulating EndMT. Changes in HDAC expression and the use of HDAC inhibitors have also been suggested as modulating the progression of atherosclerosis and other vascular diseases by regulating macrophage cholesterol efflux and vascular smooth muscle cell proliferation (28, 29). Furthermore, HDACs have been implicated in a range of additional cardiovascular diseases, including pulmonary artery hypertension (30), reperfusion injury and fibrosis (31), cardiac hypertrophy and heart failure (32), stroke (33), neointima formation (34, 35), and abdominal aortic aneurysm (36).

Based on this body of data, we speculated that changes in histone PTMs and HDACs may govern EndMT. Furthermore, we hypothesized that by modulating EndMT, HDACs may participate in the maintenance of endothelial cell homeostasis and also in the progression of atherosclerosis and plaque instability.

## Results

### EndMT is associated with deacetylation of histone H3 lysine residues and increased HDAC9 expression.

We used a previously published method (12) of EndMT induction on 8 lines of human coronary artery endothelial cells (HCAECs) (from 8 different donors). We used HCAECs because these are the most relevant cells for coronary artery disease (CAD). A combination of TGF-β2 plus H_2_O_2_ was more potent in inducing EndMT in HCAECs than either agent alone and resulted in significant downregulation of endothelial genes, including zona occludens 2 (*ZO2*), intercellular adhesion molecule 2 (*ICAM2*), occludin, and endothelial nitric oxide synthase (*eNOS*) (Supplemental Figure 1A; supplemental material available online with this article; https://doi.org/10.1172/JCI131178DS1), with upregulation of mesenchymal genes transgelin (*SM22α*), fibroblast activation protein (*FAP*), versican, and calponin (Supplemental Figure 1B). EndMT induction also resulted in an upregulation of TGF-β pathway–associated transcription factors *SNAIL* and *SLUG* (Supplemental Figure 1C). These results are similar to what was seen in previously reported findings using venous endothelial cells (12).

**Figure 1:**
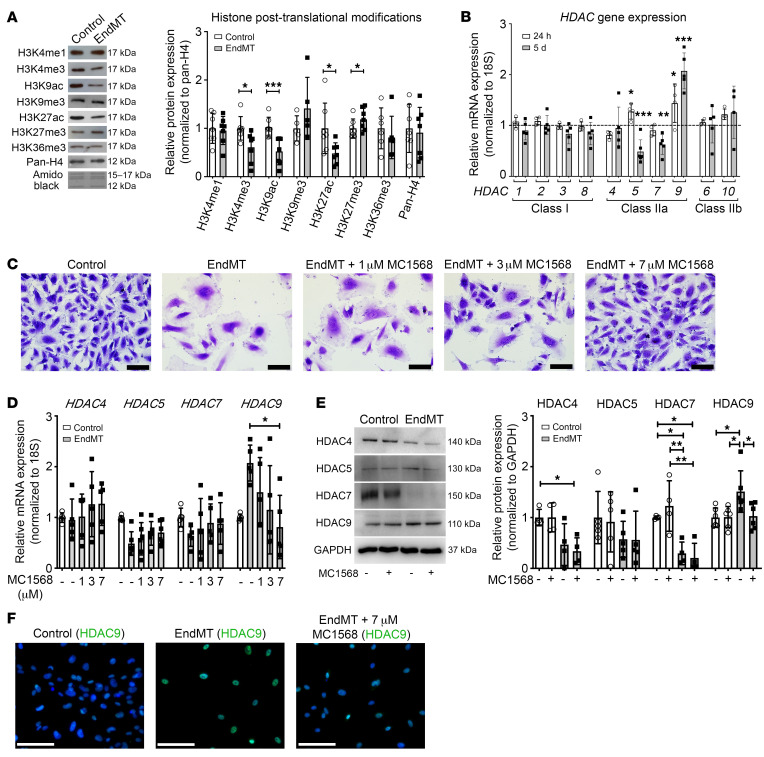
EndMT is associated with histone deacetylation and increased HDAC9 expression, which is ameliorated by class IIa HDAC inhibitor MC1568. (**A**) Representative Western blots for histone PTMs after 5 days of EndMT induction in HCAECs with densitometric analysis demonstrating changes in histone acetylation and methylation. *n* = 5–7. (**B**) qRT-PCR showing time-dependent changes of canonical HDACs after 24-hour and 5-day EndMT induction (TGF-β2 plus H_2_O_2_) in HCAECs. Graph is representative of fold change relative to vehicle-treated control cells normalized to 1 (dashed line). *n* = 4–6. (**C**) Images of HCAECs after 5-day EndMT induction with or without increasing doses of MC1568. Scale bars: 100 μm. (**D**) qRT-PCR analysis showing mRNA expression of class IIa HDACs (HDAC4, -5, -7, and -9) after 5-day EndMT induction in HCAECs, with a dose-dependent effect of MC1568 on *HDAC9* gene expression. *n* = 5–6. (**E**) Representative Western blots and densitometry measurements of class IIa HDACs after 5-day EndMT induction with or without MC1568 in HCAECs, with the graph representing fold change relative to vehicle-treated controls. *n* = 4–6. (**F**) Immunofluorescence staining of control HUVECs with anti-HDAC9 antibody and after 5-day EndMT induction with or without 7 μM MC1568. Scale bars: 100 μm. *P ≤ 0.05; **P ≤ 0.01; ***P ≤ 0.001. Analyses performed using unpaired Student’s *t* test (**A**), 1-way ANOVA (**B** and **D**), and 2-way ANOVA (**E**).

To study relevant epigenetic effects, we first sought to characterize broad patterns of histone PTMs during EndMT. Chromatin was extracted from HCAECs after EndMT induction and screened for the abundance of common histone PTMs compared with control cells. This demonstrated that EndMT is associated with a global decrease in histone PTMs that are associated with gene activation, specifically H3K4me3, H3K9ac, and H3K27ac, with a concomitant increase in H3K27me3 (Figure 1A). Since HDACs are responsible for the deacetylation of histone residues, gene expression levels of class I and II HDACs were measured using quantitative real-time PCR (qRT-PCR) at 24 hours and 5 days after EndMT induction. The mRNA expression of class IIa HDACs was most significantly altered during EndMT, with a 2-fold increase in *HDAC9* and a reduction in *HDAC5* and *-7* (Figure 1B). Conversely, the expression of class I and IIb HDACs remained unchanged during EndMT, suggesting that class IIa HDACs may play a role in EndMT. Because we observed a generalized reduction of histone acetylation with EndMT (Figure 1A) and HDAC9 was the only HDAC to fit this pattern (increased HDAC9 expression with EndMT; Figure 1B), we reasoned that HDAC9 may play an important function in EndMT and elected to pursue its potential role in this process.

### Class IIa HDAC inhibition prevents HDAC9 upregulation and H3 deacetylation during EndMT.

To investigate the potential role of class IIa HDACs in EndMT, we used the chemical HDAC inhibitor MC1568 (37, 38) during EndMT induction. MC1568 is a selective inhibitor of class IIa HDACs and is reported to have greater than 170-fold selectivity over class I HDACs, including HDAC1 (38–40). In addition, as well as inhibiting class IIa HDAC activity, MC1568 has been shown to inhibit class IIa HDAC expression (41, 42).

EndMT caused changes in HCAEC morphology and reduced cell confluency, which were dose dependently prevented by increasing concentrations of MC1568 (Figure 1C). With respect to the effect of MC1568 on class IIa HDAC levels, MC1568 dose dependently prevented the EndMT-induced increase in *HDAC9* mRNA (Figure 1D). MC1568 also prevented the EndMT-associated increase in HDAC9 protein expression, as shown by both Western blotting (Figure 1E) and immunofluorescence staining (Figure 1F). MC1568 treatment during EndMT did not alter the mRNA expression of any other class IIa HDACs (Figure 1D) or HDACs from class I and class IIb (Supplemental Figure 1, D and E). Interestingly, unlike HDAC9 and in the opposite direction of the generalized reduction of histone acetylation with EndMT (Figure 1A), protein expression of HDAC4 and -7 was significantly reduced following EndMT induction, and this remained unchanged in the presence of MC1568 (Figure 1E). Addition of 7 μM MC1568 prevented the EndMT-associated reduction in H3K9 and H3K27 acetylation and also prevented the increase in H3K27 methylation (Supplemental Figure 1, F and G). This dose of 7 μM MC1568 was chosen based on a dose response in which this was the highest dose of MC1568 for which no cell death was observed (results not shown).

Class IIa HDACs are known to shuttle between the nucleus and cytoplasm depending on their activation state and whether or not they are targeting histones or cytoplasmic proteins. Therefore, subcellular fractionation was performed to investigate the localization of HDAC9 during EndMT and with MC1568. We identified both nuclear and cytoplasmic HDAC9 and showed that the EndMT-induced increase in HDAC9 protein primarily occurs in the cytoplasmic fraction and is prevented by MC1568 (Supplemental Figure 2A). Since histone acetylation can also be altered via HATs, we evaluated the protein expression of 2 key HATs: P300 and P300/CBP-associated factor (PCAF). Both P300 and PCAF protein expression were significantly reduced with EndMT, and while the reduction of PCAF was prevented with MC1568, P300 remained significantly downregulated (Supplemental Figure 2B). This suggests that while the deacetylation associated with EndMT is likely due to increased HDAC9, restoration of acetylation in the presence of the class IIa HDAC inhibitor can be due to a lack of combined HDACs as well as the action of HATs.

### Class IIa HDAC inhibition affects EndMT-associated gene and protein expression.

**Figure 2:**
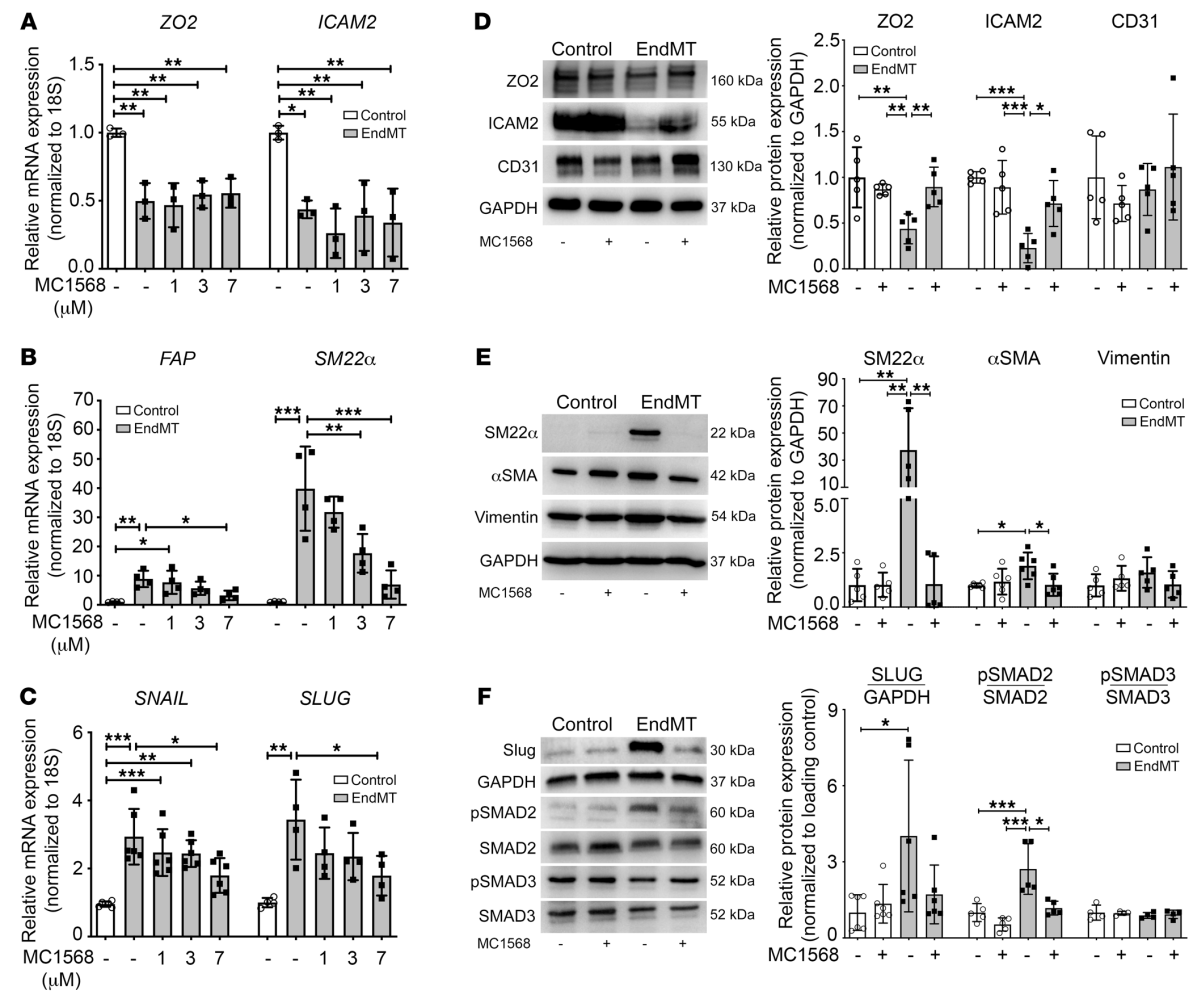
Class IIa HDAC inhibition suppresses EndMT-associated changes in mRNA and protein levels. (**A**–**C**) qRT-PCR analysis of endothelial (*ZO2*, *ICAM2*; *n* = 3), mesenchymal (*SM22α*, *FAP*; *n* = 4), and TGF-β pathway (*SNAIL*, *SLUG*; *n* = 4–6) transcript levels after 5-day EndMT induction with or without increasing doses of MC1568 in HCAECs. (**D** and **E**) Representative Western blots and densitometry measurements of endothelial (ZO2, ICAM2, CD31) and mesenchymal proteins (SM22α, αSMA, Vimentin) in HCAECs after 5-day EndMT with or without 7 μM MC1568 treatments compared with vehicle-treated controls. *n* = 5–6. (**F**) Representative Western blots and densitometry measurements of TGF-β–associated transcription factor proteins (SLUG, pSMAD2, pSMAD3) in HCAECs after 5-day EndMT with or without 7 μM MC1568 treatment compared with vehicle-treated controls. *n* = 4–6. *P ≤ 0.05; **P ≤ 0.01; ***P ≤ 0.001. Analyses performed using 1-way ANOVA (**A**–**C**) and 2-way ANOVA (**D**–**F**).

Since MC1568 prevented many of the EndMT-induced changes in epigenetic regulators of acetylation, the effect of MC1568 on classical EndMT-associated genes and proteins was then examined. While we observed no effect on endothelial gene expression with MC1568 in the setting of EndMT (Figure 2A), MC1568 prevented EndMT-associated increases in *SM22α* and *FAP* mRNA expression in a dose-dependent manner (Figure 2B) and also regulated the TGF-β pathway by inhibiting EndMT-associated increases in *SNAIL* and *SLUG* (Figure 2C). Interestingly, despite no effect on endothelial gene expression, MC1568 prevented the EndMT-associated decline in endothelial proteins ZO2 and ICAM2 (Figure 2D). In terms of mesenchymal protein upregulation with EndMT, MC1568 abolished the EndMT-associated increase in SM22α and αSMA protein expression with a modest effect on vimentin (Figure 2E). MC1568 also prevented EndMT-associated increases in SLUG protein and the increased phosphorylation of SMAD2 (Figure 2F). Furthermore, MC1568 prevented the EndMT-associated increase in FAP^+^ endothelial cells, as assessed by flow cytometry (Supplemental Figure 2C). Collectively, these results demonstrate that class IIa HDACs are responsible for regulating many of the widespread molecular and genetic changes that occur during EndMT.

Given that all observed EndMT-associated changes were reversed by MC1568 except for *ZO2* and *ICAM2* mRNA expression (Figure 2A), ChIP–quantitative PCR (ChIP-qPCR) was performed to elucidate the underlying epigenetic mechanisms. ChIP for H3K27ac with subsequent qPCR showed that at the promoter regions of both *ZO2* and *ICAM2*, H3K27ac enrichment was dramatically decreased upon induction of EndMT, which remained unrestored in the presence of MC1568 (Supplemental Figure 2D). As a positive control for ChIP-qPCR, H3K27ac enrichment at the *FAP* promoter was also examined and exhibited an increase during EndMT induction, which was partially prevented by MC1568. ChIP-qPCR was also performed for the enrichment of H3K9ac at the *ZO2* and *ICAM2* promoters, which showed that H3K9ac plays no role in regulating the expression of these 2 genes, as the enrichment of H3K9ac remained unchanged in all conditions (Supplemental Figure 2E). In contrast, H3K9ac enrichment was significantly increased at the promoter region of *FAP* (*P* ≤ 0.01, Supplemental Figure 2E) and modestly prevented by MC1568. Together, these results confirm that MC1568 does not regulate the gene expression of endothelial markers *ICAM2* and *ZO2*. However, these data demonstrate that increased mesenchymal (*FAP*) gene expression during EndMT arises in the setting of enhanced promoter acetylation, which is partially reversible by class IIa HDAC inhibition with MC1568.

To extend these findings, i.e., that class IIa HDAC inhibition with MC1568 can prevent mesenchymal, but not endothelial, gene expression changes during EndMT, we employed a different model of EndMT-induction using TGF-β2+IL-1β in HUVECs for further broad validation. Consistent with our findings in HCAECs, EndMT increased *HDAC9* expression, which was prevented by the addition of MC1568 (Supplemental Figure 3A). Similarly, MC1568 was able to reverse EndMT-induced gene expression of *SLUG* (Supplemental Figure 3B) and mesenchymal genes (Supplemental Figure 3, F–H), while having no effect on endothelial gene expression (Supplemental Figure 3, C–E).

### Class IIa HDAC inhibition prevents EndMT-induced changes in endothelial cell phenotype.

Given that class IIa HDAC inhibition with MC1568 blocked EndMT-induced gene and protein changes in HCAECs in vitro, we next explored its effect on the endothelial phenotype. EndMT induction resulted in a decrease in cell proliferation, as evidenced by reduced cell density in culture and BrdU cell proliferation assay, which was partly rescued by MC1568 (Supplemental Figure 4, A and B). There was no difference in endothelial cell apoptosis with EndMT, and there was a nonsignificant reduction in apoptosis due to MC1568, although the proportion of cells undergoing apoptosis was low in all conditions (Supplemental Figure 4C). We next explored tubule-forming capacity and contraction — with tubule formation being a classical endothelial trait that is reduced with EndMT, while contraction is a mesenchymal cell trait that is enhanced by EndMT — although it is noted that these assays may not be entirely specific to EndMT (1, 43). Consistent with transition to a mesenchymal-like phenotype, after induction of EndMT, HCAECs exhibited decreased ability to form tubules (Supplemental Figure 4D) and increased contractility (Supplemental Figure 4E). Class IIa HDAC inhibition using MC1568 partially rescued both of these traits (Supplemental Figure 4, D and E). These results indicate that, in addition to preventing gene and protein changes, class IIa HDAC inhibition also inhibits aspects of the endothelial phenotypic and functional changes that arise due to EndMT.

### Exogenous HDAC9 overexpression promotes EndMT-related changes in gene and protein expression.

To confirm that HDAC9 is responsible for the effect of class IIa HDAC inhibition on EndMT and that HDAC9 has a causal relationship with EndMT-associated changes in endothelial and mesenchymal gene and protein expression, HUVECs were transfected with a plasmid (pCMV-3tag6-HDAC9) containing full-length, Flag-tagged *HDAC9* or empty plasmid as a control. Transfection caused an increase in transient, exogenous *HDAC9* mRNA (Supplemental Figure 5A) and protein expression (Supplemental Figure 5B), which remained largely cytoplasmic (Supplemental Figure 5C). Increased HDAC9 resulted in increased expression of *FAP*, *αSMA*, vimentin, and *SLUG*, with no effect observed on *ZO2* and *ICAM2* mRNA levels (Supplemental Figure 5A). Consistent with our hypothesis that HDAC9 promotes EndMT, exogenous HDAC9 caused a decrease in the protein expression of ZO2 and ICAM2, which was prevented by MC1568 (Supplemental Figure 5D). Collectively, these results provide further evidence that HDAC9 regulates the molecular changes associated with EndMT.

### Ex vivo endothelial-specific Hdac9 knockout attenuates EndMT.

**Figure 3:**
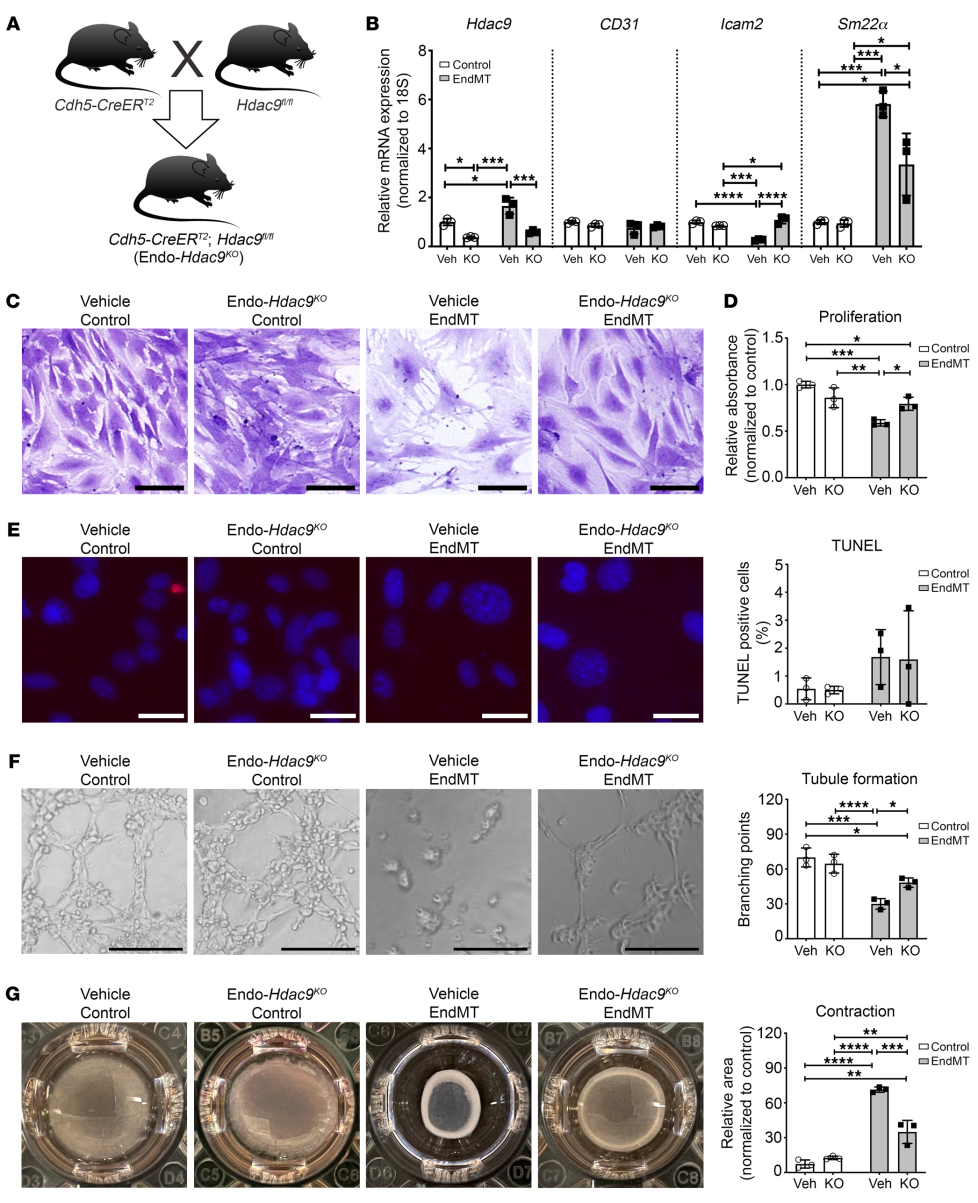
Ex vivo endothelial-specific *Hdac9* knockout attenuates EndMT-associated changes in endothelial cell function in MPLECs. (**A**) Breeding and generation of Endo-*Hdac9^KO^* mice for obtaining MPLECs. (**B**) Expression of *Hdac9*, *CD31*, *Icam2*, and *Sm22α* in MPLECs with (KO; i.e., ex vivo 4-OH tamoxifen treatment) and without (Veh; vehicle treatment) knockout of *Hdac9* with or without EndMT assessed by qRT-PCR. These groups are identical for all subsequent panels in this figure. (**C**) Crystal violet staining showing changes in cell numbers and density. Scale bars: 100 μm. (**D**) To assess proliferation, MPLECs were incubated with BrdU, followed by spectrophotometric quantification. Data are represented as fold change compared with vehicle-treated control cells. (**E**) Representative images and quantification of TUNEL assay to detect apoptosis on MPLECs with or without EndMT induction. Scale bars: 30 μm. (**F**) Tubule formation of MPLECs with or without EndMT assessed by plating cells onto Matrigel and incubating for another 4 hours. Tubule branch points were imaged and quantified. Scale bars: 100 μm. (**G**) Contraction assay showing changes in relative unoccupied area (normalized to a completely unoccupied well) for MPLECs with or without EndMT induction. For this figure, lungs from *n* = 4 male Endo-*Hdac9^KO^* mice were pooled to derive MPLECs. Apart from (crystal violet staining) (**C**), *n* = 3 for all analyses as biological replicates. *P ≤ 0.05; **P ≤ 0.01; ***P ≤ 0.001; ****P ≤ 0.0001. All analyses performed using 2-way ANOVA.

To further investigate the role of HDAC9 in EndMT, we created a mouse model with endothelial-specific *Hdac9* knockout (*Cdh5-CreER^T2^;Hdac9^fl/fl^*, henceforth Endo-*Hdac9^KO^*) (Figure 3A). To first characterize the effects of endothelial-specific *Hdac9* knockout at the cellular level, we cultured mouse primary lung endothelial cells (MPLECs) from Endo-*Hdac9^KO^* mice and induced *Hdac9* knockout ex vivo by treatment with 4-hydroxytamoxifen (4-OH tamoxifen). Compared with what occurred in excipient-treated control cells, *Hdac9* expression was reduced by approximately 65% in tamoxifen-treated cells (Figure 3B). Treatment with tamoxifen and ex vivo knockout of *Hdac9* had no effect on baseline levels of *CD31*, *Icam2*, or *Sm22α* gene expression. However, upon induction of EndMT, the increase in *Sm22α* and decrease in *Icam2* levels were rescued in cells with knockout of *Hdac9* (Figure 3B). Using the same functional assays as shown in Supplemental Figure 4 for HCAECs in vitro treated with MC1568, we observed almost identical results with MPLECs ex vivo with and without knockout of *Hdac9*. Specifically, EndMT induction resulted in morphological changes and a decrease in cell proliferation that was rescued by knockout of *Hdac9* (Figure 3, C and D). There was no difference in endothelial cell apoptosis either with EndMT or due to *Hdac9* knockout (Figure 3E). Similarly again to what is shown in Supplemental Figure 4, after induction of EndMT, MPLECs exhibited decreased ability to form tubules and showed increased contractility. Knockout of *Hdac9* partially rescued both these mesenchymal traits (Figure 3, F and G, respectively). Collectively, these findings using MPLECs with or without *Hdac9* deletion confirm that HDAC9 plays a key role in governing EndMT. Moreover, the nearly identical findings shown in Supplemental Figure 4 (HCAECs undergoing EndMT with or without class IIa HDAC inhibition with MC1568) versus those in Figure 3 (MPLECs undergoing EndMT with or without *Hdac9* knockout) corroborate this hypothesis.

### Endothelial-specific Hdac9 knockout is associated with reduced EndMT and a favorable plaque phenotype in atherosclerosis-prone mice.

**Figure 4:**
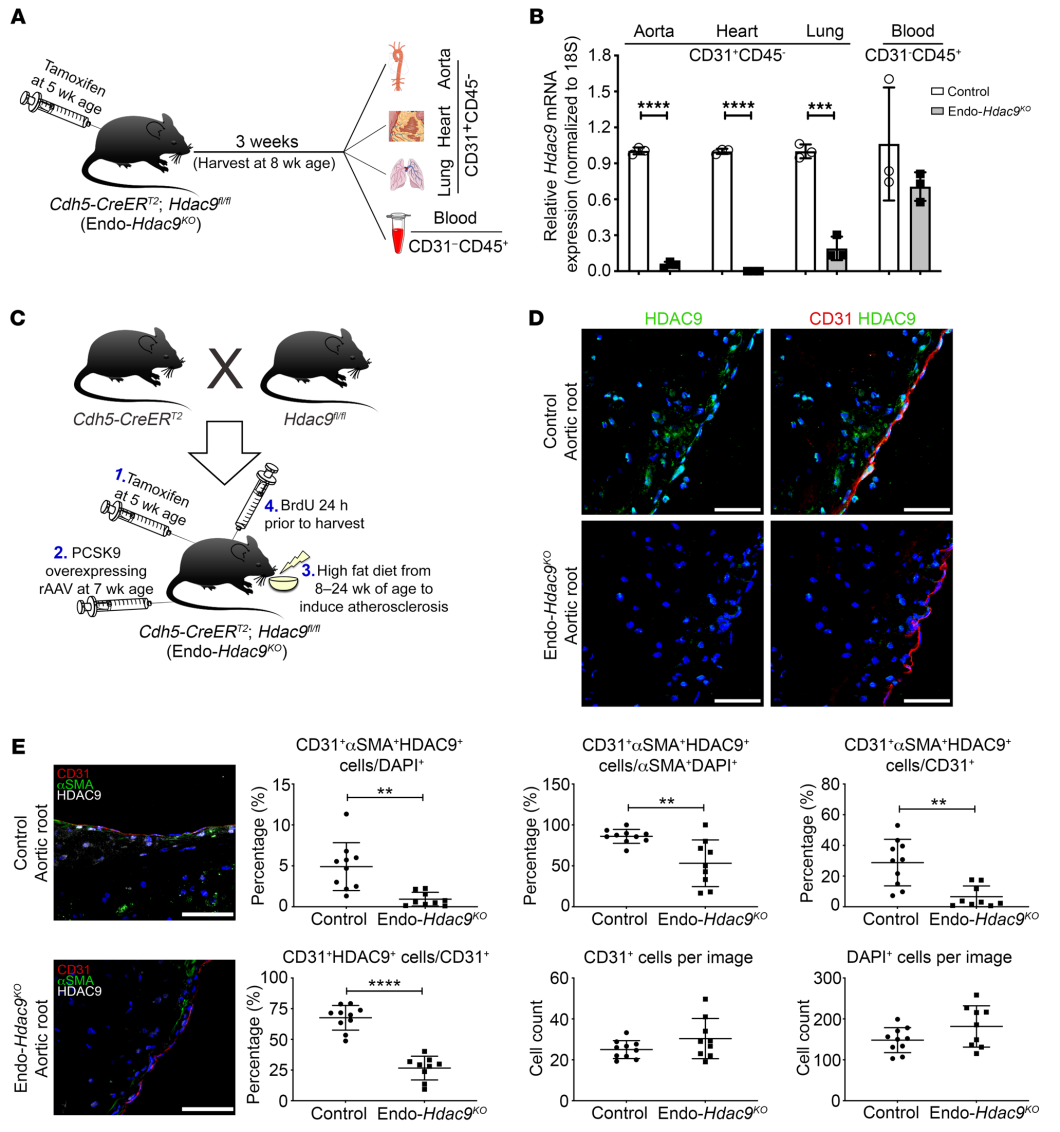
In vivo establishment and validation of Endo-*Hdac9^KO^* mouse model. All comparisons in this figure are using endothelial-specific *Hdac9* knockout mice (Endo-*Hdac9^KO^*) versus littermate controls (*Hdac9^fl/fl^*). All mice received tamoxifen. (**A**) For *Hdac9* knockout validation, endothelial cells were harvested from a variety of tissues from nonatherosclerotic Endo-*Hdac9^KO^* mice or littermate controls. (**B**) *Hdac9* knockout validation: qRT-PCR analysis of the expression levels of *Hdac9* in CD31^+^CD45^–^ endothelial cells from the aorta, heart, and lungs and CD31^–^CD45^+^ leukocytes from blood in Endo-*Hdac9^KO^* mice compared with littermate controls 3 weeks after tamoxifen administration. *n* = 3. (**C**) Breeding and generation of atherosclerotic Endo-*Hdac9^KO^* mouse model. (**D**) Representative immunofluorescence staining images for HDAC9- (green), CD31- (red), and DAPI-stained nuclei (blue) in plaques from the aortic root. (**E**) Representative immunofluorescence staining images for αSMA- (green), CD31- (red), HDAC9- (white), and DAPI-stained nuclei (blue) in aortic root plaques and quantification. Scale bars: 50 μm. *n* = 10 controls versus *n* = 9 Endo-*Hdac9^KO^* mice. **P ≤ 0.01; ***P ≤ 0.001; ****P ≤ 0.0001. All analyses performed using unpaired Student’s *t* test except CD31^+^αSMA^+^HDAC9^+^ cells/CD31^+^ (**E**), for which Mann-Whitney *U* test was used.

In view of the above data indicating that knockout of HDAC9 inhibits EndMT as well as prior knowledge that EndMT is associated with atherosclerotic plaque burden (11) and an unstable plaque phenotype (12, 13), we investigated to determine whether HDAC9 modulates EndMT in vivo and thereby affects atherosclerosis. We leveraged our Endo-*Hdac9^KO^* mouse model (*Cdh5-CreER^T2^;Hdac9^fl/fl^*) and used Cre-negative littermates as controls (*Hdac9^fl/fl^*). To activate Cre-mediated *Hdac9* deletion, all mice (*Cdh5-CreER^T2^;Hdac9^fl/fl^* and *Hdac9^fl/fl^* littermate controls) received a course of tamoxifen injections beginning at 5 weeks of age. In murine tissues harvested from these mice 3 weeks after tamoxifen injections, we confirmed the effectiveness of in vivo *Hdac9* deletion in sorted endothelial cells (CD31^+^CD45^–^) from the aorta, heart, and lungs of Endo-*Hdac9^KO^* mice, whereas circulating CD31^–^CD45^+^ leukocytes showed similar levels of *Hdac9* in both groups (Figure 4, A and B).

To induce atherosclerosis in Endo-*Hdac9^KO^* mice and their littermate controls, we administered a single dose of proprotein convertase subtilisin/kexin type 9–encoding (PCSK9-encoding) recombinant adeno-associated viral vector at 7 weeks of age and then fed mice a high-fat diet from 8 weeks of age for 16 weeks. Tissues were harvested at 24 weeks of age (Figure 4C). Compared with controls, Endo-*Hdac9^KO^* mice showed no differences in total body weight, lipid levels, or hematological cell counts (Supplemental Figure 6, A–D). Diastolic blood pressure differed marginally between groups in 8-week-old mice, but there was no subsequent difference between these same groups for diastolic blood pressure at 16 or 24 weeks of age (Supplemental Figure 6E). There was no difference between groups for systolic blood pressure at any time point (Supplemental Figure 6E).

We next performed immunofluorescence staining followed by confocal microscopy to assess HDAC9 expression patterns in the atherosclerotic aorta. In control mice, we identified clear endothelial expression of HDAC9, which was almost undetected in Endo-*Hdac9^KO^* mice (Figure 4D). Furthermore, while there was no difference in either the total number of cells or number of CD31^+^ endothelial cells, compared with plaques from control mice, plaques from Endo-*Hdac9^KO^* mice showed a marked reduction in the proportion of CD31^+^ endothelial cells that coexpressed HDAC9 (CD31^+^HDAC9^+^) and also a marked reduction in the proportion of CD31^+^HDAC9^+^ cells that appeared to be undergoing EndMT, as represented by concurrent expression of αSMA (CD31^+^HDAC9^+^αSMA^+^) (Figure 4E).

**Figure 5:**
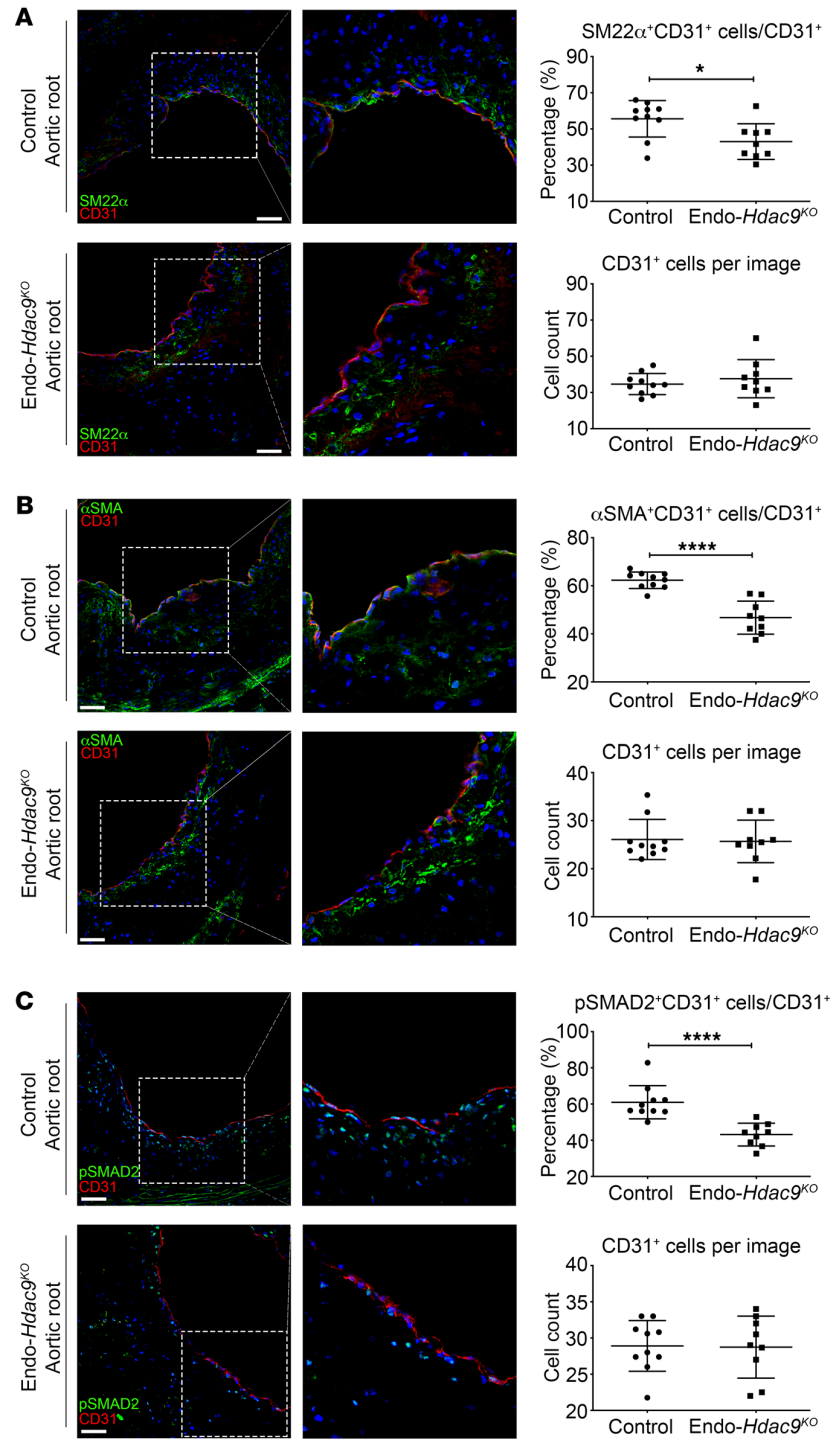
Endothelial-specific *Hdac9* knockout reduces EndMT in vivo. All comparisons in this figure were generated using aortic root sections from Endo-*Hdac9^KO^* mice versus littermate controls, and all mice received tamoxifen. (**A**) Representative immunofluorescence staining images for SM22α- (green), CD31- (red), and DAPI-stained nuclei (blue), quantification of SM22α^+^CD31^+^ copositive cells over total CD31^+^ cells, and total number of CD31^+^ cells per image. (**B**) Representative immunofluorescence staining images for αSMA- (green), CD31- (red), and DAPI-stained nuclei (blue), quantification of copositive cells over total CD31^+^ cells, and total number of CD31^+^ cells per image. (**C**) Representative immunofluorescence staining images for pSMAD2- (green), CD31- (red), and DAPI-stained nuclei (blue), quantification of copositive cells over total CD31^+^ cells, and total number of CD31^+^ cells per image. Scale bars: 50 μm. Right panels are digital enlargements of the original adjacent images. *n* = 10 controls versus *n* = 9 Endo-*Hdac9^KO^* mice. *P ≤ 0.05; ****P ≤ 0.0001. All analyses performed using unpaired Student’s *t* test except the following, for which Mann-Whitney *U* test was used: SM22α^+^CD31^+^ cells/CD31^+^ cells (**A**); CD31^+^ cells per image (**B**); and pSMAD2^+^CD31^+^ cells/CD31^+^ cells (**C**).

We also assessed the extent of EndMT on the basis of copositive staining of the endothelial marker CD31 with either SM22α, αSMA, or pSMAD2 as mesenchymal markers. We found that the proportion of cells undergoing EndMT (SM22α^+^CD31^+^ or αSMA^+^CD31^+^ or pSMAD2^+^CD31^+^ copositive cells) was significantly reduced in atherosclerotic lesions from 3 different vascular areas (aortic root, ascending aorta, aortic arch) in Endo-*Hdac9^KO^* mice compared with controls (Figure 5 and Supplemental Figure 7). Specifically, there was up to a 45% decrease in the proportion of cells undergoing EndMT in Endo-*Hdac9^KO^* mice in the aortic root, ascending aorta, and aortic arch, despite similar numbers of CD31^+^ endothelial cells (Figure 5 and Supplemental Figure 7) and CD68^+^ macrophages (Supplemental Figure 6F).

**Figure 6:**
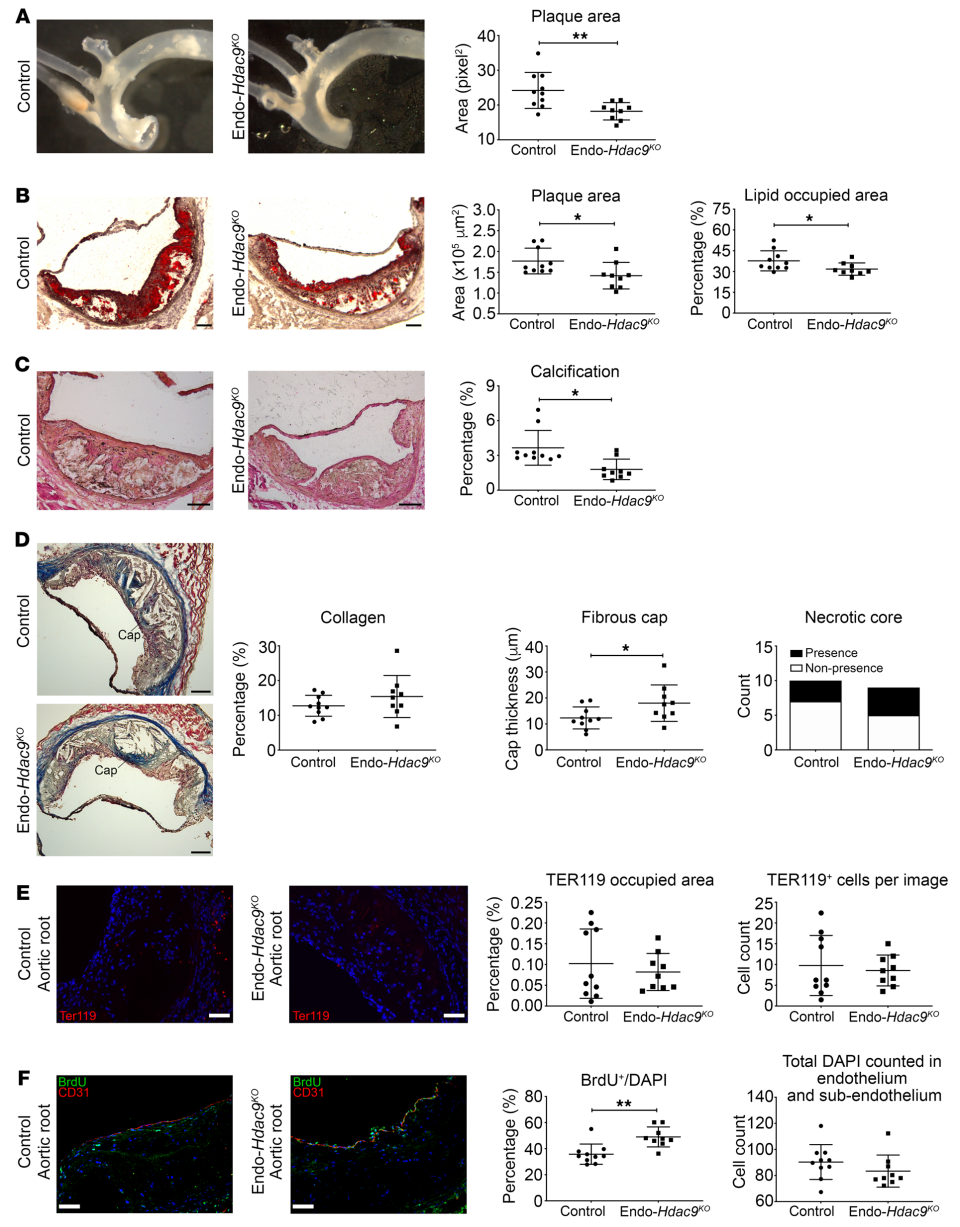
Endothelial-specific *Hdac9* knockout reduces atherosclerosis and enhances plaque stability in vivo. All comparisons shown are using Endo-*Hdac9^KO^* mice versus littermate controls, and all mice received tamoxifen. (**A**) Representative images of aorta before embedding into OCT and en face analysis of aortic plaque. (**B**) Representative images of the aortic root with staining using oil red O, with quantification of total plaque area and plaque lipid content (red stained area as a percentage of total plaque area). (**C**) Representative images of aortic root sections using Von Kossa stain (black represents calcification), with quantification of calcification. (**D**) Representative aortic root images stained with Masson’s trichrome with quantification of collagen content, fibrous cap thickness, and presence/nonpresence of necrotic core (blue, collagen; pink, macrophages and cardiac muscle). (**E**) Representative immunofluorescence staining images in aortic root plaques for TER119- (red, erythrocyte marker) and DAPI-stained nuclei (blue) and quantification. (**F**) Representative immunofluorescence staining images of aortic root plaques for BrdU- (green), CD31- (red), and DAPI-stained nuclei (blue) in the endothelial and subendothelial layers, and quantification. Scale bars: 100 μm (**B**–**D**); 50 μm (**E** and **F**). *n* = 10 controls versus *n* = 9 Endo-*Hdac9^KO^* mice for all panels. *P ≤ 0.05; **P ≤ 0.01. All analyses performed using unpaired Student’s *t* test except the following, for which Mann-Whitney *U* test was used: plaque area (**B**), calcification (**C**), and both analyses in **F**. In addition, presence or nonpresence of necrotic core (**D**) was analyzed using Fisher’s exact test.

We next turned to evaluate the atherosclerotic burden and phenotype. En face analysis of thoracic aortas demonstrated that Endo-*Hdac9^KO^* mice had a relative 25% decrease in aortic plaque area compared with control mice (Figure 6A). Consistent with this, oil red O staining indicated reduced plaque area in Endo-*Hdac9^KO^* mice compared with controls in both the aortic root and arch (Figure 6B and Supplemental Figure 8, A and B). Furthermore, plaque lipid content was significantly reduced in the aortic root and ascending aorta of Endo-*Hdac9^KO^* mice compared with controls (Figure 6B and Supplemental Figure 8, A and B). Calcification was also significantly reduced in plaques from the aortic root of Endo-*Hdac9^KO^* mice (Figure 6C), although not in the ascending aorta or aortic arch (Supplemental Figure 8, C and D). In addition, by Masson’s trichrome staining of aortic root sections, while the extent of collagen and presence of necrotic cores were similar between Endo-*Hdac9^KO^* mice and controls, there was a significant increase in fibrous cap thickness in Endo-*Hdac9^KO^* mice (Figure 6D). While there were no differences in these plaque features in the ascending aorta (Supplemental Figure 8E), in contrast, in aortic arch plaques, there was a significant increase in plaque collagen content and fibrous cap thickness in Endo-*Hdac9^KO^* mice (Supplemental Figure 8F). Intra-plaque hemorrhage was not different between Endo-*Hdac9^KO^* mice and controls (Figure 6E and Supplemental Figure 9, A and B). Finally, consistent with our ex vivo data (Figure 3D), cell proliferation in the endothelial and subendothelial layers was increased in Endo-*Hdac9^KO^* mice compared with controls (Figure 6F and Supplemental Figure 9, C and D). Collectively, these findings indicate that in the setting of atherosclerosis, the absence of *Hdac9* in endothelial cells is associated with reduced EndMT, reduced atherosclerotic burden, and a more favorable plaque phenotype.

### Class IIa HDAC inhibition reduces EndMT during atherosclerosis and leads to a more favorable plaque phenotype.

**Figure 7:**
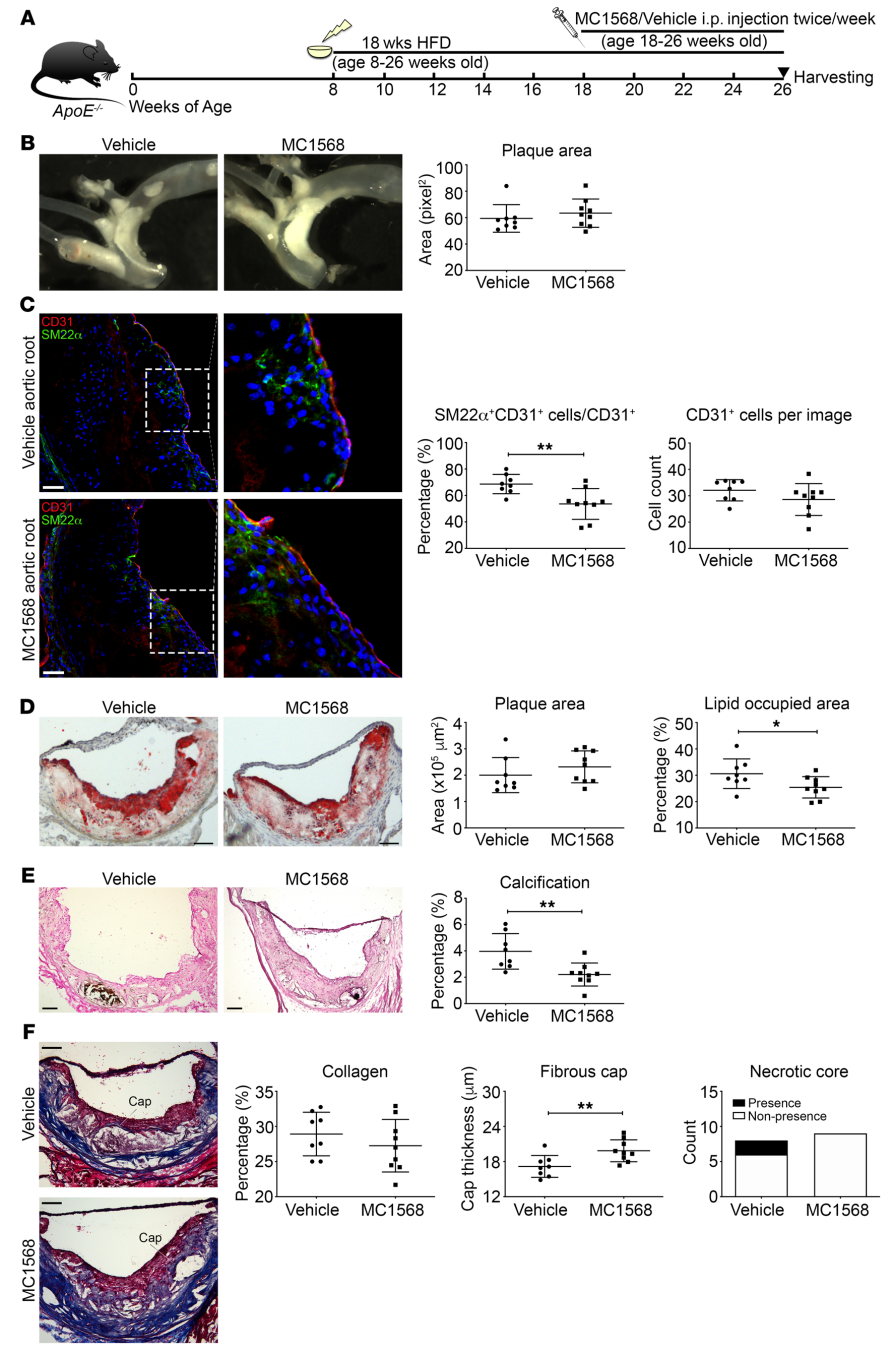
Systemic administration of a class IIa HDAC inhibitor reduces EndMT and increases plaque stability. (**A**) Generation of MC1568-treated atherosclerotic mouse model. (**B**) Representative images of aorta before embedding into OCT and en face analysis of plaque in aorta. (**C**) Representative images of immunofluorescence staining of aortic root sections for SM22α- (green), CD31- (red), and DAPI-stained nuclei (blue) and quantification of SM22α^+^CD31^+^-costained cells over total CD31^+^ cells and the total number of CD31^+^ cells per image. (**D**) Representative images of the aortic root with staining using oil red O, with quantification of total plaque area and plaque lipid content. (**E**) Representative images of aortic root sections using Von Kossa stain (black represents calcification), with quantification of calcification. (**F**) Representative aortic root images stained with Masson’s trichrome with quantification of collagen content, fibrous cap thickness, and presence/nonpresence of necrotic core (blue, collagen; pink, macrophages and cardiac muscle). Scale bars: 50 μm (**C**); 100 μm (**D**–**F**). Right panels in **C** are digital enlargements of the original adjacent images. *n* = 8 controls (vehicle) versus *n* = 9 MC1568-treated mice for all panels. *P ≤ 0.05; **P ≤ 0.01. All analyses performed using unpaired Student’s *t* test except plaque area (**B** and **D**), for which Mann-Whitney *U* test was used. In addition, presence or nonpresence of necrotic core (**F**) was analyzed using Fisher’s exact test.

Given these findings, as a potential pathway to a clinical therapy, we evaluated the efficacy of class IIa HDAC inhibition using MC1568 in mice with established atherosclerosis. Specifically, *ApoE^–/–^* mice were placed on a high-fat diet at 8 weeks of age for 18 weeks to induce atherosclerosis. After 10 weeks of high-fat diet, when atherosclerosis was already established, mice were randomly assigned to receive MC1568 or vehicle by intraperitoneal injection for 8 weeks (until sacrifice and tissue harvest) (Figure 7A). Administration of MC1568 did not alter body weight or lipid profile compared with those in vehicle-administered control mice (Supplemental Figure 10, A and B). However, compared with controls, mice receiving MC1568 showed a reduction in total WBC count, monocyte count, and lymphocyte count. There was no difference in RBC, platelet, or neutrophil counts between groups (Supplemental Figure 10, C and D).

En face analysis of thoracic aortas demonstrated no difference in aortic plaque area in mice that received MC1568 versus vehicle (Figure 7B). EndMT in atherosclerotic plaques was again evaluated by immunofluorescence costaining and confocal imaging for CD31^+^SM22α^+^ copositive cells, which demonstrated that class IIa HDAC inhibition by MC1568 was associated with a significant reduction in the proportion of endothelial cells undergoing EndMT in vivo (Figure 7C and Supplemental Figure 10, E and F). Immunofluorescence staining also showed no difference in inflammatory cell content (Supplemental Figure 11, A and B). By histochemical staining, consistent with the en face analysis, administration of MC1568 did not affect plaque area; however, there was a significant reduction in plaque lipid content (Figure 7D and Supplemental Figure 11, C and D). Compared with what occurred in control atherosclerotic mice, MC1568 treatment was also associated with a significant reduction in plaque calcification (Figure 7E). In addition, similarly to what was seen in Endo-*Hdac9^KO^* mice, while the extent of collagen and presence of necrotic cores were similar, administration of MC1568 was associated with a significant increase in fibrous cap thickness (Figure 7F).

Collectively, in mice with established atherosclerosis, these results indicate that class IIa HDAC inhibition by systemic MC1568 administration leads to reduced EndMT and favorable changes in atherosclerotic plaque morphology.

## Discussion

There is increasing evidence that EndMT plays an important role in adult cardiovascular diseases (1, 2). While our understanding of the epigenetic regulation of EMT is substantial, the epigenetic regulation of EndMT remains relatively unexplored. Our study has shown that EndMT is associated with deacetylation of key histone residues, suggesting that histone modifiers play a major role in regulating EndMT. Further, we show that inhibition of class IIa HDACs can prevent EndMT both in vitro and in vivo. Our data also demonstrate that HDAC9 is the predominant class IIa HDAC responsible for these effects. Specifically, HDAC9 regulates key EndMT-associated molecular changes, including both gene and protein expression of endothelial and mesenchymal cell markers, and also TGF-β pathway–associated transcription factors. Furthermore, both class IIa HDAC inhibition and genetic *Hdac9* knockout reversed phenotypic endothelial cell changes that were induced by EndMT and led to favorable changes in atherosclerotic plaques, suggesting potential therapeutic benefit in the treatment of atherosclerosis.

In addition to the above key findings, our results add weight to the understanding that, although major pathways may broadly govern the general phenomena of EMT and EndMT, particular molecular aspects of EMT and EndMT are differentially regulated (1, 2, 12, 13, 44). Furthermore, Neumann et al. (18) recently noted that EndMT is associated with changes in H3K4me3 methylation of 2 key EndMT-associated genes, which is likely mediated via an interaction between the long noncoding antisense transcript of GATA6 and the epigenetic regulator LOXL2. While not a specific focus of our study, we nevertheless noted a global decrease in the protein level of H3K4me3 with EndMT (Figure 1), and the results of Neumann et al. support our finding that HDACs and epigenetic mechanisms play a key role in regulating EndMT (18).

Atherosclerosis is the most common cause of cardiovascular disease and is a chronic process involving cellular, metabolic, and inflammatory events (45, 46). EndMT was recently recognized as a significant contributor to atherosclerosis (12, 13, 47); however, the mechanisms remain to be defined. In parallel, HDAC9 has been implicated in carotid intima-media thickness (48), large-vessel ischemic stroke (48, 49), atherosclerosis (50), and atherosclerotic aortic calcification in humans (51). Potentially connecting these observations, our experiments demonstrated that *Hdac9* knockout reduces the extent of EndMT in atherosclerotic mice, correlating with decreased plaque area and a more stable plaque phenotype. Moreover, class IIa HDAC inhibition through MC1568 administration, while not altering atherosclerotic plaque size, was associated with reduced EndMT and features of increased plaque stability. The issue of whether increased fibrous cap thickness is related to reduced EndMT versus a specific effect of HDAC9 is the subject of ongoing studies in our laboratory; however, these findings are consistent with prior research showing that a reduction in EndMT is associated with a beneficial effect on atherosclerosis (13, 52) and a more stable plaque phenotype in humans (12). Collectively, we propose that these data suggest a pathological HDAC9/EndMT/atherosclerosis axis. In addition, the fact that therapy with MC1568 was able to reduce EndMT and favorably alter plaque phenotype with established atherosclerosis (after 10 weeks of high-fat feeding in *ApoE^–/–^* mice) is of translational importance, as it argues for the possibility that therapies targeting EndMT via HDAC inhibition may favorably affect atherosclerosis in humans with established disease. Moreover, the appeal of potentially using HDAC inhibitors as a therapy against atherosclerosis is supported by the fact that there are several HDAC inhibitors with US FDA approval that are in current clinical use for other indications, including the pan-HDAC inhibitor vorinostat and the more selective HDAC inhibitor romidepsin (53).

As well as EndMT and atherosclerosis, our study is also part of a growing body of literature indicating that HDACs are fundamental regulators of endothelial biology and endothelial function. HDAC3 is critical for endothelial cell survival, and an increase in HDAC3 is found in endothelial cells exposed to disturbed flow (54). Knockdown of HDAC3 exacerbates the development of atherosclerosis and causes vessel rupture. HDAC3 is also essential for endothelial cell differentiation, and aberrant HDAC3 splicing can induce EndMT through modulation of the TGF-β pathway (55). HDAC5 phosphorylation and nuclear export in endothelial cells can enhance the expression of laminar flow–related atheroprotective genes, such as *KLF-2* and *eNOS* (56). Silencing of HDAC5 is proangiogenic and increases endothelial migration, sprouting, and tubule formation by increasing FGF2 expression (57). HDAC7 is fundamental in maintaining vascular integrity by acting as a repressor of MMP10 (58). Absence of HDAC7 is embryonically lethal, with endothelial cells failing to establish cell-cell junctions, which leads to vessel dilatation and rupture (58). Therefore, although we have provided substantial evidence to indicate a major role for HDAC9 in governing EndMT, we cannot exclude the additional possibility that other HDACs may also regulate certain aspects of EndMT.

In terms of HDAC9, our study showed that an increase of HDAC9 expression has potential detrimental effects in endothelial cells, caused by facilitating the loss of cell junctions and by driving endothelial cells toward a mesenchymal phenotype. In addition to our study, others have also demonstrated that HDAC9 plays an important role in endothelial regulation. For example, HDAC9 expression is increased in endothelial cells during cerebral ischemia/reperfusion injury in rats, promoting endothelial dysfunction through an increased inflammatory response, apoptosis, and cell permeability through decreased cell-cell junction proteins (59). Furthermore, overexpression of HDAC9 increases endothelial sprouting by decreasing expression of the miRNA-17-92 cluster, which is known to have antiangiogenic activity (60). Together with our findings, these studies reinforce the fact that HDAC9 is of critical importance for governing endothelial function and pathobiology.

Beyond endothelial biology, HDAC9 has been implicated in other cell types that are important in vascular disease. In mice, it has been shown that HDAC9 suppresses macrophage cholesterol efflux and that transplantation with HDAC9-null bone marrow decreases atherosclerotic lesion size and favors an antiinflammatory macrophage phenotype (61). Increased HDAC9 expression is seen in human thoracic aortic aneurysm tissue and also in cell culture models of genetically perturbed vascular smooth muscle cells where increased HDAC9 is associated with increased MMP production and dysregulation of cytoskeletal genes (62). HDAC9 also facilitates adipocyte differentiation and maturation, while HDAC9 knockdown prevents the detrimental effects of chronic high-fat feeding by improving metabolic homeostasis (63, 64). The class IIa HDAC inhibitor MC1568 (used in this study) has been reported to effectively reduce the severity and incidence of abdominal aortic aneurysms in angiotensin II–infused *ApoE^–/–^* mice by attenuating the expression of proinflammatory markers and restoring correct organization of elastin and collagen fibers (36). These studies further demonstrate that HDAC9 plays a key role in regulating the molecular dysfunction apparent in aberrant cellular phenotypes, contributing to vascular pathology.

In conclusion, our study demonstrates a unique role for HDAC9 in contributing to vascular pathology by promoting EndMT, which is associated with increased atherosclerosis and an unstable plaque phenotype. Furthermore, we have shown that selective HDAC inhibition is effective in attenuating EndMT in vitro and in vivo. Together with other studies, our data argue strongly for the existence of a pathological HDAC9/EndMT/atherosclerosis axis and suggest that targeting of HDAC9 may be beneficial for plaque stabilization or slowing the progression of atherosclerotic disease.

## Methods

### Cell culture and treatment.

HCAECs and HUVECs (Lonza, C-2585 and C2517A, respectively) were cultured at 37°C in 5% CO_2_ in endothelial basal medium-2 supplemented with EGM-2MV BulletKit (CC-3202, Lonza) and used at passages 6–7. To induce EndMT, 70,000 cells/well in a 6-well plate or 250,000 cells in a 10 cm dish were plated and designated day 0. Complete EGM-2MV medium was changed every other day (days 1 and 3 after plating). EndMT was induced by incubating cells in 50 ng/mL recombinant human TGF-β2 (100-35B, PeproTech) and 200 mM H_2_O_2_ (H1009, MilliporeSigma) added to complete media on days 1 and 3, as described (12). For experiments using MC1568 (16265, Cayman), cells were treated with 1, 3, or 7 μM MC1568, 30 minutes prior to the first EndMT induction and then daily for the duration of EndMT. Cells were harvested after 5 days of treatment. For EndMT induction using TGF-β2+IL-1β, HUVECs were treated with 10 ng/mL TGF-β2 and 1 ng/mL IL-1β daily for 7 days, and as indicated, 7 μM MC1568 was added 30 minutes prior to the addition of cytokines.

MPLECs were obtained from Endo-*Hdac9^KO^* mice (*Cdh5-CreER^T2^;Hdac9^fl/fl^*). For each batch of MPLECs, 4 Endo-*Hdac9^KO^* mice were anesthetized by intraperitoneal injection and perfused with 10 mL ice-cold HBSS^++^ buffer (Supplemental Table 1). Lungs were removed and rinsed in RPMI 1640 medium (11875093, Thermo Fisher) with 1% antibiotic/antimycotic (15240-112, Life Technologies) before being minced using a scalpel. Minced lungs were digested in 20 mL HBSS^++^ buffer containing 0.2% collagenase A (10103586001, MilliporeSigma) for 2 hours at 37°C with constant tilting. Cells were then filtered through a 70 μm nylon filter (352350, Falcon) and washed twice before resuspension in 3 mL 0.1% BSA (A9647, MilliporeSigma) in PBS (used as 1× PBS in all experiments), which was further incubated with 2.7 μg CD31-coated (553370, BD Biosciences) Dynabeads (11035, Thermo Fisher Scientific) for 60 minutes at 4°C on a roller. Bead-bound cells were collected using a magnet, resuspended in mouse endothelial cell medium (M1168, Cell Biologics), and cultured for at least 7 days to allow adherence and initial expansion. After further expansion and at passages 2–3, to ablate *Hdac9* and induce EndMT in MPLECs, 70,000 cells/well were seeded onto a 6-well plate and designated day 0. On day 1, medium was replaced with 1 mL mouse endothelial cell medium plus 10 μM 4-OH tamoxifen (H7904, MilliporeSigma) or the same volume of excipient. 4-OH tamoxifen was reconstituted in ethanol, then diluted with peanut oil (P2144, MilliporeSigma) per the manufacturer’s instructions. On days 3 and 5, medium was replaced with mouse endothelial cell medium containing (a) 2 μM 4-OH tamoxifen or equivalent volume of vehicle; and/or (b) 50 ng/mL of recombinant mouse TGF-β2 (CK35, Bon Opus Biosciences) plus 200 mM H_2_O_2_. Cells were harvested for further study on day 7 after plating.

### Flow cytometry.

Flow cytometry was performed on HCAECs after 5-day EndMT induction, with or without treatment with MC1568 as described above. After trypsinization and washing, cells were fixed for 10 minutes in 4% PFA and then washed in PBS. Cells were then stained with anti-FAP–Alexa Fluor 647 (bs-5758R-A647, Bioss) or isotype control in PBS containing 0.5% saponin for 2 hours on ice. Cells were washed with PBS before analysis on a BD LSR II Flow Cytometer.

### Animals and treatments.

For all in vivo experiments, experimental groups comprised nearly equal numbers of male and female mice, and results are presented in aggregate (males and females combined). Sex-specific data are provided for body weight.

For *Hdac9* knockout experiments, C57BL6 *Hdac9^fl/fl^* mice (62) were crossed with C57BL6 *Cdh5-CreER^T2^* mice (65) to generate an inducible endothelial-specific *Hdac9* knockout model (*Cdh5-CreER^T2^;Hdac9^fl/fl^*). All mice (*Cdh5-CreER^T2^;Hdac9^fl/fl^* and *Hdac9^fl/fl^* littermate controls) received 2 mg tamoxifen/d for 5 consecutive days via intraperitoneal injection beginning at 5 weeks of age. To induce atherosclerosis, all mice received a single dose (100 μl volume containing 1.5 × 10^11^ virus particles/mouse) of gain-of-function (D377Y) murine PCSK9-encoding recombinant adeno-associated viral vector (rAAV-*PCSK9*) at 7 weeks of age as described (66). All mice were fed a high-fat adjusted calories diet (42% from fat, TD88137, Envigo) from 8 weeks of age for 16 weeks. At week 8 (immediately before initiation of high-fat diet), week 16, and week 24 (before intraperitoneal BrdU injection), blood pressure was measured using a CODA noninvasive mouse blood pressure system (Kent Scientific Corp.) according to the manufacturer’s instructions. Three blood pressure measurements were performed, and the mean was taken. Tissues were harvested when mice were 24 weeks of age. BrdU (ab142567, Abcam) was dissolved at 10 mg/mL in sterile PBS and was administered at 10 μl/g of body weight via intraperitoneal injection 24 hours before tissue harvesting. Genotyping of mice was validated by PCR of DNA samples at 3 to 4 weeks of age (Supplemental Table 2).

For in vivo experiments with MC1568, 8-week-old *ApoE^–/–^* mice were placed on a high-fat adjusted calories diet for 18 weeks to induce atherosclerosis. After 10 weeks of high-fat diet, mice were randomly assigned to receive MC1568 (*n* = 9) or vehicle (*n* = 8) by intraperitoneal injection for 8 weeks. MC1568 was dissolved in sterile 1% caboxymethylcellulose (419273, MilliporeSigma) in PBS and administered twice weekly at 40 mg/kg during the last 8 weeks of high-fat diet. Control mice were administered 1% carboxymethylcellulose in PBS. Tissues were harvested when mice were 26 weeks of age.

All animals were euthanized by cardiac puncture and exsanguination under isoflurane inhalation anesthesia (NDC 10019-360-40, Baxter Healthcare). Blood collected from each mouse was placed into a heparinized tube and analyzed for complete blood count (CBC). Plasma was collected for lipid panel analysis by centrifuging blood at 4°C, 1000 *g*, for 10 minutes and stored at –80°C. Exsanguinated mice were perfused with 4% paraformaldehyde (15710, Electron Microscopy Sciences) plus 0.1% glutaraldehyde in PBS at 3 mL/min via the left ventricle for 7 minutes using an infusion pump (BS-300, Braintree Scientific). The heart, aorta, and great vessels were carefully dissected from the surrounding tissues. Aortas were photographed with a Zeiss microscope (Zeiss Stemi 2000-C with AxioCam ERc 5s camera) on the anterior and posterior sides for en face analysis. The aortic root and aorta were then placed in 20% sucrose in PBS at 4°C overnight before embedding in OCT (62550-01; Electron Microscopy Sciences) and stored at –80°C. The aortic root and aorta were cryosectioned at 10 μm thickness (CM3050S, Leica) onto precleaned glass slides (12-550-19; Fisher Scientific) and stored at –80°C.

### CBC and cholesterol analysis.

CBC analysis was performed by the Comparative Pathology Laboratory at the Icahn School of Medicine at Mount Sinai using an IDEXX ProCyte Dx Hematology Analyzer (IDEXX BioResearch). Cell counts of RBCs, WBCs, platelets, monocytes, lymphocytes, and neutrophils were obtained using standard procedures according to the manufacturer’s instructions. Plasma was sent to IDEXX laboratories for chemical testing. Levels of total cholesterol, HDL cholesterol, LDL cholesterol, and triglycerides were measured according to standard procedures using a Catalyst Dx Analyzer (IDEXX BioResearch).

### FACS.

Endo-*Hdac9^KO^* mice (*Cdh5-CreER^T2^;Hdac9^fl/fl^*) and *Hdac9^fl/fl^* littermate controls received tamoxifen as described above at 5 weeks of age, and tissues were harvested at 8 weeks of age. Aortas were digested as previously described (12). Each heart was digested with 0.8 Wünsch units/mL of Liberase DL (05401160001, Roche) in 1 mL of serum-free RPMI 1640 medium at 37°C for 45 minutes. Lungs from each mouse were digested with 1 mL HBSS^++^ buffer containing 0.5% collagenase A at 37°C for 45 minutes. Blood was also obtained and treated with RBC lysis buffer (00-4333-57, eBioscience). In brief, 30 mL ice-cold RBC lysis buffer was added to 2 mL blood. After incubation for 15 minutes, the mixture was centrifuged for 8 minutes at 400 *g*. After aspirating the supernatant, another 30 mL ice-cold RBC lysis buffer was added and the pellet resuspended. After further incubation for 15 minutes, the mixture was again centrifuged for 8 minutes at 400 *g*. Each sample was finally resuspended in 900 μl RPMI 1640 buffer. Cells from all samples were stained with CD45-APC (1800-11, Fisher; 1 μg/million cells), CD31-PE (102408, BioLegend; 1 μg/million cells), and DAPI, followed by sorting using a FACS LSRII flow cytometer (BD Biosciences). qRT-PCR was performed on sorted cells to evaluate *Hdac9* levels as described below.

### Histological and immunofluorescence analysis of atherosclerotic lesions.

Studies were performed in accordance with the 2017 American Heart Association recommendations for animal atherosclerosis studies (67). Frozen sections were thawed at room temperature and rinsed in PBS to remove OCT. For all immunofluorescence staining except as described, tissues were first blocked using 20% donkey serum (D9663, MilliporeSigma) with 5% BSA in PBS. For BrdU staining, slides were incubated with prewarmed 1N HCL (1090571000, MilliporeSigma) for 45 minutes at 37°C before blocking. Sections were then incubated overnight at 4°C with primary antibodies in 3% BSA in PBS (for antibodies, see Supplemental Table 6). Isotype control slides were incubated with isotype-specific IgG with the same concentration and species as the primary antibody. Slides were then washed 3 times in PBS and incubated for 1 hour at room temperature with the appropriate secondary antibodies: anti-rat Alexa Fluor 546 at 4.0 μg/mL (catalog A11081, Invitrogen) and/or anti-rabbit Alexa Fluor 488 at 4.0 μg/mL (catalog A21206, Invitrogen). Prior to imaging, slides were washed 3 times with PBS and mounted with VECTASHIELD Antifade Mounting Medium with DAPI (H-1200, Vector Laboratories). For immunohistochemical analysis, aortic sections were stained with oil red O (ab150678, Abcam), Masson’s trichrome (HT15, MilliporeSigma), and Von Kossa stain (ab150687, Abcam) according to the manufacturer’s instructions. Presence of necrotic core was defined as a typically appearing necrotic core occupying 15% or greater of the total plaque by Masson’s trichrome staining (67). Immunohistochemical and CD45 or CD68 images were acquired using a Leica DMi8 microscope, while other immunofluorescence staining was imaged by confocal microscopy (LSM780, Zeiss). All quantification was done blinded, and data were averaged from at least 3 separate plaque sections in aortic root and 2 separate plaque sections in the ascending and arch aorta per mouse. ImageJ (NIH, version 1.52a for Windows) or Fiji software (version 2.0.0-rc-68/1.52w for Mac) was used for image analysis and quantification.

### HDAC9 staining.

For HDAC9 immunofluorescence staining of mouse samples, tissues were fixed with 4% PFA for 10 minutes and permeabilized with 0.3% Triton X-100 with PBS for another 10 minutes. Sections were then blocked with 5% BSA in antibody diluent with background-reducing components (S3022, DAKO) for 1 hour at room temperature, followed by overnight incubation at 4°C in antibody diluent with background-reducing components plus 0.1% Triton X-100 with the following primary antibodies in the indicated combinations: anti-HDAC9, anti-CD31, and/or anti-αSMA (see Supplemental Table 6). Sections were incubated with appropriate secondary antibodies diluted into antibody diluent with background-reducing components and incubated for 1 hour at room temperature. Slides were imaged by confocal microscope (LSM 780, Zeiss).

For HDAC9 immunocytochemistry staining of HUVECs, cells were first cultured under 3 conditions: control, EndMT induction, and EndMT induction plus 7 μM MC1568, as described above. HUVECs were fixed with 4% PFA for 10 minutes, followed by washing with PBS, then permeabilization with 0.3% Triton X-100 with PBS for another 10 minutes. HUVECs were next blocked for 30 minutes with 0.1% Tween-20 in 5% BSA and then incubated overnight at 4°C on a rocker with anti-HDAC9 antibody (catalog ab109446, Abcam). Cells were then washed in PBS at room temperature and incubated with anti-rabbit Alexa Fluor 488 as secondary antibody (catalog A21206, Invitrogen) in 1% BSA in PBS. Finally, cells were washed and incubated with DAPI (D3571, Invitrogen) diluted 1:1000 in PBS for 5 minutes. The DAPI-containing solution was then replaced with PBS, and cells were imaged on an inverted microscope (DMi8, Leica).

### qRT-PCR.

For cells in culture except HUVECs, shown in Supplemental Figure 3, RNA was extracted using an RNeasy Mini Kit according to the manufacturer’s instructions (74104, QIAGEN) and stored at –80°C until use. RNA quantity and quality were analyzed using a NanoDrop 2000c Spectrophotometer (Thermo Scientific). Reverse transcription was performed using the iScript cDNA Synthesis Kit (170-8891, Bio-Rad) according to the manufacturer’s instructions. The reaction conditions were 25°C for 5 minutes, 46°C for 20 minutes, 95°C for 1 minute, and finally maintained at 4°C. For cells obtained from FACS (endothelial cells or leukocytes), RNA was extracted using an RNeasy Micro Kit according to the manufacturer’s instructions (74004, QIAGEN). cDNA was synthesized and amplified using the qRT-PCR–compatible Ovation RNA-Seq System, version 2 (7102, Tecan Genomics). cDNA quantity and quality were analyzed using a NanoDrop 2000c Spectrophotometer. For all cell types except HUVECs, shown in Supplemental Figure 3, qRT-PCR was performed using the PerfeCTa SYBR Green FastMix Reaction Kit according to the manufacturer’s instructions (101414-292, VWR) and was performed at 95°C for 5 minutes, followed by 40 cycles of 95°C for 5 seconds and 60°C for 30 seconds. 18S rRNA was used as a control and gene expression analyzed using the ΔΔCt method. Primers are listed in Supplemental Tables 3 and 4.

For HUVECs shown in Supplemental Figure 3, except for *HDAC9*, qRT-PCR was performed using TaqMan Gene Expression probes. RNA isolation, reverse transcription, and qRT-PCR were performed as described in Monteiro et al. (68). For detection of *HDAC9* in HUVECs, qRT-PCR was performed using the PerfeCTa SYBR Green FastMix Reaction Kit as described above. Human ubiquitin protein C (UBC) was used for qRT-PCR normalization in Supplemental Figure 3.

### ChIP-qPCR.

Total chromatin extraction was performed as previously described (69) with the following modifications. Briefly, cells were lysed in buffer A (10 mM HEPES pH = 7.9, 10 mM KCl, 1.5 mM MgCl_2_, 0.34M sucrose, 10% glycerol, inhibitor cocktail: 1 mM DTT, 0.1% Triton X-100) and incubated for 8 minutes on ice. Nuclear pellets were obtained by centrifugation at 1,500 *g* for 5 minutes at 4°C, washed twice with buffer A, and placed into Douncing buffer (10 mM Tris, 4 mM MgCl_2_, 1 mM CaCl_2_, pH 7.5). Pellets of unfixed nuclei were processed for native-ChIP as previously described (70) starting from step 30 with the following modifications. ChIP was performed using 4 μl anti-H3K27ac antibody, 4 μl anti-H3K9ac antibody, or 4 μl anti-IgG antibody. ChIP-qPCR was performed using the Perfecta SYBR Green FastMix Reaction Kit according to the manufacturer’s instructions (101414-292, VWR). ChIP-qPCR was performed at 95°C for 5 minutes followed by 40 cycles of 95°C for 5 seconds and 60°C for 30 seconds using primers corresponding to the promoter region of each gene of interest listed in Supplemental Table 5. qRT-PCR was performed in triplicate, and the percentage of input was calculated. Data are representative of *n* = 4 independent experiments.

### Histone PTM and protein Western blotting.

For histone PTM quantification, chromatin was obtained as previously described (69) with the following modifications. Final chromatin pellets were resuspended in Laemmli sample buffer (161-0737, Bio-Rad) plus 5% β-mercaptoethanol and boiled for 5 minutes at 95°C. Histones were separated by SDS-PAGE gel electrophoresis, transferred to polyvinylidene fluoride membrane (PVDF, 88520, Thermo Fisher Scientific), and stained using amido black staining solution (A8181, MilliporeSigma) to confirm equal loading. Membranes were blocked with 5% skim milk powder in TBS-t for 1 hour at room temperature.

For protein extraction, cells were collected into RIPA buffer (89900, Thermo Scientific) with protease and phosphatase inhibitor (78441, Thermo Scientific) on ice and lysed using a BD Lo-Dose insulin syringe (14-826-79, Fisher Scientific). Protein concentration was measured using a Pierce BCA Protein Assay Kit (23255, Thermo Fisher Scientific) according to the manufacturer’s instructions using a SpectraMax M5 microplate reader (Molecular Devices). Ten micrograms of protein was loaded and separated by SDS-PAGE gel electrophoresis, transferred to PVDF membrane, and blocked in 5% BSA for 1 hour at room temperature.

Primary antibodies were incubated overnight at 4°C as indicated in Supplemental Table 6. Membranes were washed in TBS-t and incubated in goat anti-rabbit IgG (H+L) secondary antibody, HRP (diluted 1:15,000, catalog 31460, ThermoFisher Scientific), or goat anti-mouse IgG (H+L) secondary antibody, HRP (diluted 1:15,000, catalog 31430, ThermoFisher Scientific) for 1 hour at room temperature. Membranes were visualized using Immobilon Western chemiluminescence HRP substrate (WBKLS0500; EMD Millipore) and imaged on a ChemiDoc Touch Imaging System (Bio-Rad). The full, uncut gels can be found in the supplemental material. Densitometry analysis was performed using Image Lab 5.2 software from Bio-Rad.

### Cell fractionation.

Cells (5 × 10^6^) were collected and washed twice with cold PBS. The cell pellet was transferred into a prechilled microcentrifuge tube and gently resuspended and incubated for 15 minutes with 500 μL 1× hypotonic buffer (20 mM Tris-HCl 7.5, 10 mM NaCl, 3 mM MgCl_2_, with protease and phosphatase inhibitor). Then, 25 μl 10% NP40 was added and vortexed for 10 seconds. The homogenate was centrifuged at 4°C for 10 minutes at 1,200 *g*, and the supernatant (cytoplasmic fraction) was removed and stored at –80°C. The nuclear pellet was washed once with 250 μL 1× hypotonic buffer with protease and phosphatase inhibitor. The nuclear pellet was then resuspended in 50 μL RIPA buffer for 30 minutes on ice with vortexing at 10-minute intervals. The homogenate was centrifuged at 4°C for 30 minutes at 14,000 *g*. The supernatant (nuclear fraction) was stored at –80°C prior to Western blotting.

### Crystal violet and TUNEL assay.

HCAECs at a density of 18,000 cells/well or MPLECs at 25,000 cells/well were seeded onto 24-well plates. HCAECs were treated with or without EndMT and with or without MC1568 as described above. MPLECs were treated with 4-OH tamoxifen or vehicle and with or without EndMT, as described above (quantities of reagents and media were scaled to 24-well format). Cells were then fixed with ice-cold 4% PFA for 10 minutes and stained with 0.5% crystal violet (C3886, MilliporeSigma) for another 10 minutes before washing with tap water. Images were taken with an inverted microscope (DMi8, Leica). For TUNEL assay, HCAECs or MPLECs were cultured and fixed as described for the crystal violet assay. An In Situ Cell Death Detection Kit (12156792910, MilliporeSigma) was used according to the manufacturer’s instructions.

### Cell proliferation assay.

For HCAECs, cells were plated onto a 96-well plate at 3000 cells/well in replicates of 5 and EndMT induced as indicated above. After 24 hours, a BrdU Cell Proliferation ELISA Colorimetric Kit (11647229001, Roche) was used according to the manufacturer’s instructions. BrdU was applied for 10 hours, and absorbance was measured at 370 nm using a SpectraMax M5 microplate reader. The average of 5 experimental replicates was used to calculate the average proliferation rate per HCAEC line.

For MPLECs, cells were treated with 4-OH tamoxifen or vehicle for 2 days and then also with or without EndMT induction for another 2 days (4 treatment conditions in total). Cell were then trypsinized and plated onto a 96-well plate at 3000 cells/well in replicates of 3 and were cultured for a further 2 days with or without induction of EndMT (without 4-OH tamoxifen or vehicle). A BrdU Cell Proliferation Assay Kit (K306-200; BioVision) was then used according to the manufacturer’s instructions. In brief, BrdU was incubated for 4 hours. Absorbance was measured at 650 nm using a SpectraMax M5 microplate reader 25 minutes after TMB substrate was added. The average of 3 experimental replicates was used to measure proliferation for each biological replicate.

### Tubule-formation assay.

For HCAECs, cells underwent EndMT induction for 5 days with or without MC1568. MPLECs were treated with 4-OH tamoxifen or vehicle and with or without EndMT, as described above. Cells were then trypsinized, counted, and plated onto growth factor–free Matrigel (CB-40230A, Fisher Scientific) coated onto a 96-well plate at 6000 cells/well. Cells were replaced into the incubator for 4 hours before imaging. Two images/well were captured at ×10 magnification using phase-contrast microscopy. Tubule-branching points were counted using ImageJ software.

### Contraction assay.

For HCAECs, cells underwent EndMT induction for 5 days with or without MC1568. MPLECs were treated with 4-OH tamoxifen or vehicle and with or without EndMT, as described above. Thereafter, cells were trypsinized and resuspended in complete medium at 1 × 10^6^ cells/mL. Cell suspensions were mixed 1:4 with a collagen gel working solution according to the manufacturer’s protocol (CBA-201, Cell Biolabs) and plated onto 48-well plates using 250 μL/well and allowed to set for 1 hour. After the collagen was set, 500 μL serum-free medium was loaded into each well for 1 hour to serum starve the cells before replacing 400 μL of serum-free medium with complete medium plus lysophosphatidic acid (LPA) (catalog 3854, R&D Systems; contraction initiator, final concentration at 10 μM), and the gels were then released. Gels were imaged after 24 hours and contraction measured by calculating gel area using ImageJ software.

### Exogenous HDAC9 overexpression.

HUVECs were seeded into 10 cm dishes. After 24 hours, cells were serum starved for 1 hour prior to transfection with Lipofectamine 3000 transfection reagent according to the manufacturer’s instructions (Thermo Fisher Scientific; L3000008). Cells were transfected with Flag-tagged full-length *HDAC9* cDNA construct (71) cloned in a pCMV-3tag6 vector (Agilent Technologies) or empty vector as a control. Cells were harvested at 24 hours, 48 hours, and 72 hours after transfection and tested for exogenous HDAC9 expression. *HDAC9* mRNA was detected by qRT-PCR with primers shown in Supplemental Table 3, and HDAC9 protein was detected by Western blotting using an anti-HDAC9 primary antibody and anti-Flag antibody, as listed in Supplemental Table 6. Exogenous HDAC9 localization was detected using anti-Flag primary antibody after cells were fixed in 4% paraformaldehyde.

### Statistics.

No outliers or data points were excluded from this study. For all HCAEC experiments, 3 to 8 different donors were used as biological replicates and all data were acquired through a minimum of 3 independently performed experiments. For all HUVEC and MPLEC experiments, a minimum of 3 independent experiments were performed. For in vitro and ex vivo data, unpaired Student’s *t* test, 1-way ANOVA, or 2-way ANOVA with post hoc Tukey’s multiple comparison test was used as stated. For in vivo data, we first tested each data set using the Shapiro-Wilk test to assess for normality of distribution. For normally distributed in vivo data, we applied an unpaired Student’s *t* test. For in vivo data that were not normally distributed, we applied a Mann-Whitney *U* test. A single exception for the in vivo data was for considering the presence or nonpresence of necrotic core, where, due to the categorical nature of these data, Fisher’s exact test was used. The specific test applied for each comparison is stated in each figure legend. Statistical analyses were performed using Prism 8 or 9, and a 2-sided *P* value of less than 0.05 was considered significant. All data are presented as mean ± SD.

### Study approval.

All mice were housed in the animal facility at Icahn School of Medicine at Mount Sinai. Animal experiments and protocols were approved by Institutional Animal Care and Use Committee of Icahn School of Medicine at Mount Sinai.

## Author contributions

LL and YX conceived, performed, and analyzed experiments, prepared figures, and cowrote the manuscript. BV performed histochemical staining and helped with mouse genotyping and harvesting. NC, VDE, MPS, and LM helped with and provided advice on in vitro and in vivo experiments and data. VP performed ChIP-qPCR experiments. DKC and PAG performed *HDAC9* pCMV-3tag6 vector cloning. AC and AHB performed EndMT in vitro experiments in HUVECs and analyzed these data. MMB and JFB provided rAAV-*PCSK9* and advised on in vivo experiments. HWK and NLW provided pCMV-3tag6-*HDAC9* and *Hdac9^fl/fl^* mice and advised on the use of these resources. SK, MB, VF, and JLMB conceived and supervised several analyses and assisted with manuscript editing. EB provided antibodies and advice for histone PTM quantification, assisted with designing experiments, and helped edit the manuscript. JCK conceived the study, supervised experiments and data analysis, and cowrote the manuscript. All authors edited the manuscript.

## Supplementary Material

Supplemental data

## Version 1 08/02/2021

Electronic publication
