# Supplementary material for: Histone deacetylase 9 promotes endothelial-mesenchymal transition and an unfavorable atherosclerotic plaque phenotype
Source: J Clin Invest. 2021 Aug 2;131(15):e131178. doi: 10.1172/JCI131178 (PMC8321575; doi:10.1172/JCI131178)
Supplement: Supplemental data [file jci-131-131178-s017.pdf]

SUPPLEMENTAL DATA

Histone deacetylase 9 promotes endothelial to mesenchymal transition and an unfavorable atherosclerotic plaque phenotype

Laura Lecce,[#] Yang Xu,[#] Bhargavi V'Gangula, Nirupama Chandel, Venu Pothula, Axelle Caudrillier, Maria Paola Santini, Valentina d'Escamard, Delaine K. Ceholski, Przemek A. Gorski, Lijiang Ma, Simon Koplev, Martin Mæng Bjørklund, Johan L.M. Björkegren, Manfred Boehm, Jacob Fog Bentzon, Valentin Fuster, Ha Won Kim, Neal L. Weintraub, Andrew H. Baker, Emily Bernstein, Jason C Kovacic.

# Equal contribution

SUPPLEMENTAL TABLES

Table S1. HBSS^{++} buffer for mouse primary lung endothelial cell (MPLEC) tissue culture.

Reagent	Company	Catalog number	Volume
HBSS	Thermo Fisher Scientific	14175095	243.5ml
Calcium Chloride	Millipore Sigma	21115	0.5ml
Magnesium Sulfate	Millipore Sigma	M3409	1ml
1M Hepes	Thermo Fisher Scientific	15630080	5ml

Table S2. Primer list for mouse genotyping.

Gene	Forward Primer	Reverse Primer
Hdac9$^{fl/fl}$	ACCAAATACCAGTCCATGGAAG	GACCTTTACCACCTCATAGAGAC
Cdh5-CreER^{T2}	GCATTACCGGTCGATGCAACGA GTGATGAG	GAGTGAACGAACCTGGTCGAAATCA GTGCG

Table S3. Human primer list for RT-qPCR.

Gene	Forward Primer	Reverse Primer
ZO2	AGGAGGTGGGAGAGAGCAGT	CTTGTGCGTTTTGATTGGAA
ICAM2	CCTTGTCACCTTGTCACCAA	CAGAGAGGGGAACGGACTAA
Occludin	TGCATGTTCGACCAATGC	AAGCCACTTCCTCCATAAGG
eNOS	GTGGCTGTCTGCATGGACCT	CCACGATGGTGACTTTGGCT
SM22α	GTTCCAGACTGTTGACCTCTTT	CTGCGCTTTCTTCATAAACC
FAP	CAAGTGGCAAGTGGGAGGCCA	TGGGGATGCCTGGGCCGTAG
Versican	GGTGCACTTTGTGAGCAAGA	TTCGTGAGACAGGATGCTTG
Calponin	GGCCAGCATGGCGAAGACGAAA	TGTGCCCAGCTTGGGGTCGT
Vimentin	GGCTCAGATTCAGGAACAGC	AGCCTCAGAGAGGTCAGCAA
αSMA	CAAGTGATCACCATCGGAAAT	GACTCCATCCCGATGAAGGA
SNAIL	GCACGGCCTAGCGAGTGGTT	GGGCTGCTGGAAGGTAAACTCTGG
SLUG	AACTCACACGGGGGAGAAGCCT	CAGTGTGCTACACAGCAGCCAGA
SMAD2	AGTGCCCCGACACACCGAGA	TCTGCTGGAGAGCCTGTGTCC
SMAD3	TCAAGAAGACGGGGCAGCTGGA	GCACCAACACAGGAGGTAGAACTGG
HDAC1	CCTGAGGAGAGTGGCGATGA	GTTTGTCAGAGGAGCAGATCGA
HDAC2	GCTCTCAACTGGCGGTTCAG	AGCCCAATTAACAGCCATATCAG
HDAC3	GCTTCCACTCCGAGGACTAC	CTTGTTGTTCAGCTGGGTTG
HDAC4	AGACTTGTGGCACGAGAAGG	GGTGCTCAGGTAGGGAGTGA
HDAC5	AGCTCCTGTTCGCTGAGTTC	TGCTTCTCCAGCTCTTCCTG
HDAC6	AGATACTGGCGCAGCTTACG	GTTAGTCTGGCCTGGAGTGG
HDAC7	CAGGGGCTGACAAAGAAGAA	AGGCTGTTCTCTGGTTCCAA
HDAC8	AAGCCTAAAGTGGCCTCCAT	TGTGATCGTAGCCCCTCCTA
HDAC9	CAGCAACAAATCCAGAAGCA	GTTCTCTGCGATGCCTCTCT
HDAC10	GTTTCCTGAGCTGCATCTTG	GCTGCCCTCTCAGGTACATC
18S	TTTCGGAACTGAGGCCATGA	GCAAATGCTTTCGCTCTGGTC

Specific to Figure S3 only, the following TaqMan™ primers were used. For RT-qPCR of *HDAC9* in Figure S3, the above forward and reverse primer sequences were used.

Gene	TaqMan™ Assay ID (Applied Biosystems)
UBC	Hs01871556_s1
CD31	Hs01065282_m1
CDH5	Hs00901465_m1
SLUG	Hs00161904_m1
αSMA	Hs00426835_g1
COL1A1	Hs00164004_m1
eNOS	Hs01574659_m1
SM22α	Hs01038777_g1

Table S4. Mouse primer list for RT-qPCR.

Gene	Forward Primer	Reverse Primer
CD31	ATCGGCAAAGTGGTCAAGAG	AATGGCAATTATCCGCTCTG
Icam2	ACGGTCTCAACTTTTCCTGCC	TGCATCGGCTCATAGACTTCAA
Sm22α	ACGACCAAGCCTTCTCTGCCTCA	CCACGATCTGGGCGGCCTAC
Hdac9	AGCAGATCCAGAAGCAGCTC	TGCTCCAGTTTCTGCTCCTT
18S	TTTCGGAACTGAGGCCATGA	GCAAATGCTTTCGCTCTGGTC

Table S5. Primers used for ChIP-PCR.

Gene	Forward Primer	Reverse Primer
ZO2 set-a (Promoter 1)	GGGTAGAGGAGCTGACAT	GAATTTGCTGGGCTTCTTTC
ZO2 set-b (Promoter 1)	AAGCCCAGCAAATTCCTCTT	GGGAGACCTTTGTCACTGGA
ZO2 set-c (Promoter 2)	AGCTCACTCCAGGTCTC	ACTGGAGCAAGCAGGTC
ICAM2 set-a	CACGGCTTATTCAGTCTTCTC	TGGAGCTAAATGAGGCTAGT
ICAM2 set-b	CCTGTGGGCTCCAAGAA	GACTGAATAAGCCGTGAACTG
FAP set-a	TGCCATCTGGTGAAATTTAGT	CATTCTTCCAAAGGTCTGCT

Table S6. Primary antibody list.

Antibody	Company	Catalog number	Dilution or concentration used	Experimental technique
H3K4me1	Abcam	ab8895	1μg/ml	WB
H3K4me3	EMD Millipore	05-745R	1:10,000	WB
H3K9ac	EMD Millipore	07-352	1:5000<br>4μl	WB<br>ChIP
H3K9me3	EMD Millipore	07-442	1:1000	WB
H3K27ac	Abcam	ab4729	1μg/ml<br>4μl	WB<br>ChIP
H3K27me3	EMD Millipore	07-449	1:2,500	WB
H3K36me3	EMD Millipore	07-549	1:1000	WB
Pan-H4	EMD Millipore	05-858	1:10,000	WB
HDAC4	Santa Cruz Biotechnology	sc-365093	0.4μg/ml	WB
HDAC5	Novus Biologicals	NBP2-22152	2μg/ml	WB
HDAC7	Cell Signaling Technology	33418	1:1000	WB
HDAC9	Novus Biologicals<br>Abcam	NBP2-03993<br>ab109446	0.5μg/ml<br>11.98μg/ml	WB<br>ICC and IF
GAPDH	Abcam	ab181602	21μg/ml	WB
ZO2	Santa Cruz Biotechnology	sc-11448	0.13μg/ml	WB
ICAM2	Bethyl Laboratories	A304-348A	1:1500	WB
CD31	Abcam<br>BD Biosciences	ab9498<br>550274	0.15μg/ml<br>0.31μg/ml	WB<br>IF
SM22α	Abcam	ab14106	0.7μg/ml<br>7μg/ml	WB<br>IF
αSMA	Abcam<br>Abcam<br>Sigma Aldrich	ab5694<br>ab5694<br>F3777	0.2μg/ml<br>2μg/ml<br>1:100	WB<br>IF (CD31-αSMA)<br>IF (CD31-HDAC9-αSMA)
Vimentin	Cell Signaling Technology	5741	1:1000	WB
FAP	Bioss	bs-5758R-A647	2μl/1x10^6 cells	FC
pSMAD2	Cell Signaling Technology<br>Cell Signaling Technology	3108<br>3108	1:1000<br>1:100	WB<br>IF
SMAD2	Cell Signaling Technology	5339	1:1000	WB

pSMAD3*	Abcam	ab51451	1μg/ml	WB
SMAD3	Cell Signaling Technology	9513	1:1000	WB
SLUG	Novus Biologicals	NBP2-27182	0.2μg/ml	WB
Flag	Sigma-Aldrich	F3165	1μg/ml<br>0.1μg/ml	WB<br>ICC
P300	Santa Cruz Biotechnology	sc-32244	0.8μg/ml	WB
PCAF	Santa Cruz Biotechnology	sc-13124	2.2μg/ml	WB
CD45	Abcam	ab10558	10μg/ml	IF
CD68	Bio-Rad	MCA1957	10μg/ml	IF
TER119	Invitrogen	14-5921-82	5μg/ml	IF
BrdU	Novus Biologicals	NBP2-14890	3.9μg/ml	IF

WB = Western Blotting, ChIP = Chromatin Immunoprecipitation, FC = Flow Cytometry, ICC = Immunocytochemistry, IF = Immunofluorescence staining (mouse samples). *Note that at the time of experimentation, the Abcam antibody ab51451 was marketed only as an anti pSMAD3 antibody. It was later marketed as anti pSMAD1/3/5.

SUPPLEMENTAL FIGURE LEGENDS

Figure S1. TGF-β2 + H_2O_2 is most potent in inducing EndMT in HCAECs, and the effect of MC1568 on Class I and IIb HDACs and histone acetylation. A-C, Change in mRNA expression of endothelial genes (Panel A - *ZO2, ICAM2, Occludin and eNOS*), mesenchymal genes (Panel B - *SM22α, FAP, Versican and Calponin*) and TGF-β-associated transcription factors (Panel C - *SNAIL, SLUG, SMAD2 and SMAD3*) in HCAECs after 5 days of TGF-β2 ± H_2O_2 treatment compared with vehicle-treated controls by qRT-PCR. Graphs indicate fold-change in mRNA relative to vehicle-treated controls, n=4-8. D and E, mRNA expression of Class I and IIb HDACs in HCAECs after 5-day EndMT induction ± MC1568 compared with untreated controls, as analyzed by qRT-PCR, n=4-5. F and G, Representative western blots and densitometry measurements of H3K9ac, H3K9me3, H3K27ac, H3K27me3 in HCAECs during 5-day EndMT ± MC1568 compared with vehicle-treated controls, n=6. *$p \leq 0.05$, **$p \leq 0.01$, ***$p \leq 0.001$. Analyses performed using one-way ANOVA in A-E and two-way ANOVA in F-G.

Figure S2. Cellular localization of HDAC9, and the effect of EndMT ± MC1568 on HATs, FAP protein expression and the regulation of histone acetylation. A, Cell fractionation followed by western blotting and densitometry analysis for HDAC9 in HCAECs after EndMT ± MC1568. GAPDH was used to normalize cytoplasmic fraction loading, pan-histone 3 was used to normalize nuclear fraction loading. “C” stands for control; n=3. B, Protein expression and densitometry analysis of histone acetyltransferases P300 and PCAF in HCAECs, n=4-5. C, Flow cytometry showing the percentage of FAP^+ HCAECs after 5-day EndMT induction ± MC1568 compared with untreated controls, n=4. D-E, ChIP qRT-PCR analysis representing the % input detected by qRT-PCR of *ZO2*, *ICAM2* and *FAP* promoter DNA following ChIP for H3K27ac and H3K9ac respectively, n=4. All ChIP-qPCR results were normalized to appropriate IgG isotype controls which were present in every ChIP experiment and which resulted in an expected IgG value below 0.1. *$p \leq 0.05$, **$p \leq 0.01$, and ***$p \leq 0.001$. Analyses performed using one-way ANOVA in A, D and E, and two-way ANOVA in B-C.

Figure S3. MC1568 prevents *HDAC9* expression and changes in mesenchymal genes in an alternate model of EndMT using TGF-β2 + IL-1β treatment in HUVECs. A, qRT-PCR analysis of *HDAC9* gene expression in HUVECs after 7 days of TGF-β2 + IL-1β treatment ± MC1568 compared with untreated controls. B, Gene expression of *SLUG*, C-E, endothelial genes, *CD31, eNOS* and *CDH5* and; F-H, mesenchymal genes *αSMA*, *COL1A1* and *SM22α* in HUVECs. Graphs indicate fold-change in mRNA relative to vehicle-treated controls, n=3-9 independent experiments. *$p \leq 0.05$, **$p \leq 0.01$, ***$p \leq 0.001$ and ****$p \leq 0.0001$. All analyses performed using two-way ANOVA.

Figure S4. In vitro class IIa HDAC inhibition attenuates EndMT-associated changes in endothelial cell function in HCAECs. A, Crystal violet staining showing changes in cell numbers and density after 5 days of EndMT induction (with TGF-β2 + H_2O_2) in HCAECs ± MC1568. Scale bars=100µm. B, Following 24h of EndMT induction, HCAECs were incubated for a further 10h with BrdU, followed by spectrophotometric quantification. Proliferation data are represented as fold-change normalized to vehicle-treated control cells, n=8. C, Representative images and quantification of TUNEL assay on HUVECs ± EndMT induction, n=3. Scale bars=30µm. D, Tubule formation assessed after EndMT induction in HCAECs by plating cells onto Matrigel and incubating for another 4h before assessment. Tubule branch points were imaged and quantified, n=4. Scale bars=100µm. E, Contraction assay showing changes in relative unoccupied area (normalized to a completely unoccupied well) for HCAECs after 5-days EndMT induction ± MC1568, n=4. *$p \leq 0.05$, **$p \leq 0.01$, ***$p \leq 0.001$ and ****$p \leq 0.0001$. All analyses performed using two-way ANOVA.

Figure S5. Exogenous *HDAC9* overexpression promotes EndMT. A, mRNA expression of *HDAC9*, mesenchymal and endothelial genes after exogenous overexpression of *HDAC9* following transfection with a full-length *HDAC9* expressing plasmid or empty plasmid as control, n=4. B, Western blotting for Flag and HDAC9 confirming expression of exogenous HDAC9 24h and 48h after transfection. GAPDH was used as loading control. C, Immunocytochemistry for Flag showing predominantly cytoplasmic and peri-nuclear protein expression of exogenous HDAC9 in HUVECs after transfection with a Flag-tagged, *HDAC9* overexpressing plasmid (pCMV-3tag6-*HDAC9*). Scale bars=50µm. D, Representative western blots and densitometry measurements of endothelial proteins (ZO2, ICAM2) in HUVECs transfected with Flag-tagged *HDAC9* overexpressing (pCMV-3tag6-*HDAC9*) plasmid or empty plasmid ± MC1568, n=4-5. *p≤0.05, **p≤0.01, and ***p≤0.001. All analyses performed using one-way ANOVA.

Figure S6. Body weights, blood counts, lipid panels and blood pressure of Endo-*Hdac9*^{KO} mice are unaltered compared to littermate controls. Endo-*Hdac9*^{KO} mice were compared with littermate controls (*Hdac9*$^{fl/fl}$). All mice underwent an identical course of tamoxifen injections and induction of atherosclerosis. Blood was drawn for these comparisons immediately after weighing, at the time of sacrifice and tissue harvest. A, Comparison of final body weight for male and female mice showing no difference between Endo-*Hdac9*^{KO} mice and controls. B, Comparison of plasma lipid levels showing no difference between Endo-*Hdac9*^{KO} mice and controls. TG, triglycerides; HDLc, high density lipoprotein cholesterol; LDLc, low density lipoprotein cholesterol. C-D, Comparison of complete blood counts showing no difference between Endo-*Hdac9*^{KO} mice and controls (n=6 mice per group). RBC, red blood cells; WBC, white blood cells; Mono, monocytes; Lympho, lymphocytes; Neutro, neutrophils. E, Systolic and diastolic blood pressure measured with CODA® system at 8-, 16-, and 24- weeks of age. F, Representative immunofluorescence images of aortic root sections with staining of CD68$^+$ cells (red), with nuclei counterstained using DAPI (blue). Graph shows analysis of the area occupied by CD68$^+$ cells as a percentage of total plaque area. Scale bars=100µm. Unless otherwise stated, n=10 controls versus n=9 Endo-*Hdac9*^{KO} mice. *p≤0.05. All analyses performed using unpaired Student's t-test except the following where Mann-Whitney test was used: both analyses in panel A; LDL-c and HDL-c in panel B; systolic and diastolic blood pressure at week 8 in panel E; and diastolic blood pressure at week 24 in panel E.

Figure S7. Endothelial-specific *Hdac9* knockout reduces EndMT in different aortic areas in vivo. All comparisons in this figure were generated using sections from the ascending aorta and aortic arch from Endo-*Hdac9*^{KO} mice versus littermate controls, and all mice received tamoxifen. This figure corresponds to Figure 5 where equivalent images and data are presented from the aortic root. A-B, Representative immunofluorescence staining images for SM22α (green), CD31 (red) and DAPI stained nuclei (blue), quantification of SM22α$^+$CD31$^+$ co-positive cells over total CD31$^+$ cells, and the total number of CD31$^+$ cells per image. C-D, Representative immunofluorescence staining images for αSMA (green), CD31 (red) and DAPI stained nuclei (blue), and quantification of co-positive cells over total CD31$^+$ cells and the total number of CD31$^+$ cells per image. E-F, Representative immunofluorescence staining images for pSMAD2 (green), CD31 (red) and DAPI stained nuclei (blue), and quantification of co-positive cells over total CD31$^+$ cells and the total number of CD31$^+$ cells per image. Scale bars=50µm. n=10 controls versus n=9 Endo-*Hdac9*^{KO} mice. **p≤0.01, ***p≤0.001 and ****p≤0.0001. All analyses performed using unpaired Student's t-test.

Figure S8. Endothelial-specific *Hdac9* knockout reduces atherosclerosis and enhances plaque stability in vivo. All comparisons in this figure are using Endo-*Hdac9*^{KO} mice versus littermate controls, and all mice received tamoxifen. This figure corresponds to Figure 6, B–D,

where equivalent images and data are presented from the aortic root. A-B, Representative images with staining using Oil Red O, with quantification of total plaque area and plaque lipid content (red stained area as a percentage of total plaque area) for the ascending aorta and aortic arch. C-D, Representative images of ascending aorta and aortic arch sections using Von Kossa stain (black represents calcification), with quantification of calcification. E-F, Representative ascending aorta and aortic arch images stained with Masson's trichrome with quantification of collagen content, fibrous cap thickness, and presence or non-presence of necrotic core. n=10 controls versus n=9 Endo-*Hdac9*^{KO} mice for all panels. *$p \leq 0.05$ and ***$p \leq 0.001$. All analyses performed using unpaired Student's t-test except the following where Mann-Whitney test was used: plaque area in panel A; calcification in panel D; and collagen content in panels E and F. In addition, presence or non-presence of necrotic core in panels E and F was analyzed using Fisher's exact test.

Figure S9. Endothelial-specific *Hdac9* knockout reduces atherosclerosis and enhances plaque stability in vivo. As per Figure S8, all comparisons in this figure are using Endo-*Hdac9*^{KO} mice versus littermate controls, and all mice received tamoxifen. This figure corresponds to Figure 6E and 6F, where equivalent images and data are presented from the aortic root. A-B, Representative immunofluorescence staining images in ascending aorta and aortic arch plaques for TER119 (red – an erythrocyte marker) and DAPI stained nuclei (blue), and quantification. C-D, Representative immunofluorescence staining images in ascending aorta and aortic arch plaques for BrdU (green), CD31 (red) and DAPI stained nuclei (blue) in the endothelial and sub-endothelial layers, and quantification. Scale bars=50μm. n=10 controls versus n=9 Endo-*Hdac9*^{KO} mice for all panels. **$p \leq 0.01$ and ****$p \leq 0.0001$. All analyses performed using unpaired Student's t-test except both analyses in panels A and B where Mann-Whitney test was used.

Figure S10. Effects of systemic MC1568 administration on EndMT and mouse body weight, lipid panel, blood counts and EndMT in atherosclerotic plaques. *ApoE* null mice were fed HFD for 18 weeks and received MC1568 or vehicle alone via twice-weekly intraperitoneal injections for the last 8 weeks of HFD. This figure corresponds to Figure 7. Blood was drawn for these comparisons immediately after weighing, at the time of sacrifice and tissue harvest. A, Comparison of final body weight for male and female mice showing no difference between MC1568 (n=9) and vehicle (n=8) treated mice. B, Comparison of plasma lipid levels showing no difference between MC1568 (n=9) and vehicle (n=8) treated mice; TG, triglycerides; HDLc, high density lipoprotein cholesterol; LDLc, low density lipoprotein cholesterol. C-D, Comparison of complete blood counts including red blood cells (RBC), platelets, white blood cells (WBC), monocytes (Mono), lymphocytes (Lympho) and neutrophils (Neutro) in vehicle (n=8) and MC1568 (n=5) treated mice. E-F, Representative images of immunofluorescence staining of ascending aorta and aortic arch sections for SM22α (green), CD31 (red) and DAPI stained nuclei (blue), quantification of SM22α^{+}CD31^{+} co-stained cells over total CD31^{+} cells, and the total number of CD31^{+} cells per image. Scale bars=50μm. n=8 each for groups of vehicle and MC1568 injected mice. *$p \leq 0.05$, **$p \leq 0.01$ and ***$p \leq 0.001$. All analyses performed using unpaired Student's t-test except the following where Mann-Whitney test was used: platelets in panel C, lymphocytes in panel D, and CD31^{+} cells per image in panel E.

Figure S11. Plaque leukocyte numbers and lipid content in additional vascular beds of MC1568-administrated *ApoE*$^{-/-}$ mice. *ApoE* null mice were fed HFD for 18 weeks and received MC1568 or vehicle alone via twice-weekly intraperitoneal injections for the last 8 weeks of HFD. Frozen sections from vehicle or MC1568 treated mice were stained as indicated. This figure corresponds to Figure 7. A, Representative immunofluorescence aortic root images from vehicle (n=8) or MC1568 (n=9) treated mice stained for CD45^{+} cells (green), with nuclei counterstained using DAPI (blue). Graph represents the area of CD45^{+} cells as a percentage of total plaque area. B, Representative immunofluorescence aortic root images from vehicle (n=8) or MC1568 (n=9)

treated mice of $CD68^+$ cells (red), with nuclei counterstained using DAPI (blue). Graph represents the area of $CD68^+$ cells as a percentage of total plaque area. C-D, Representative images of the ascending aorta and aortic arch from vehicle (n=8) or MC1568 (n=8) treated mice with staining using Oil Red O, with quantification of total plaque area and plaque lipid content. Scale bars: A-B=100μm; C-D=50μm. *$p \le 0.05$. All analyses performed using unpaired Student's t-test.

Supplemental Figure 1

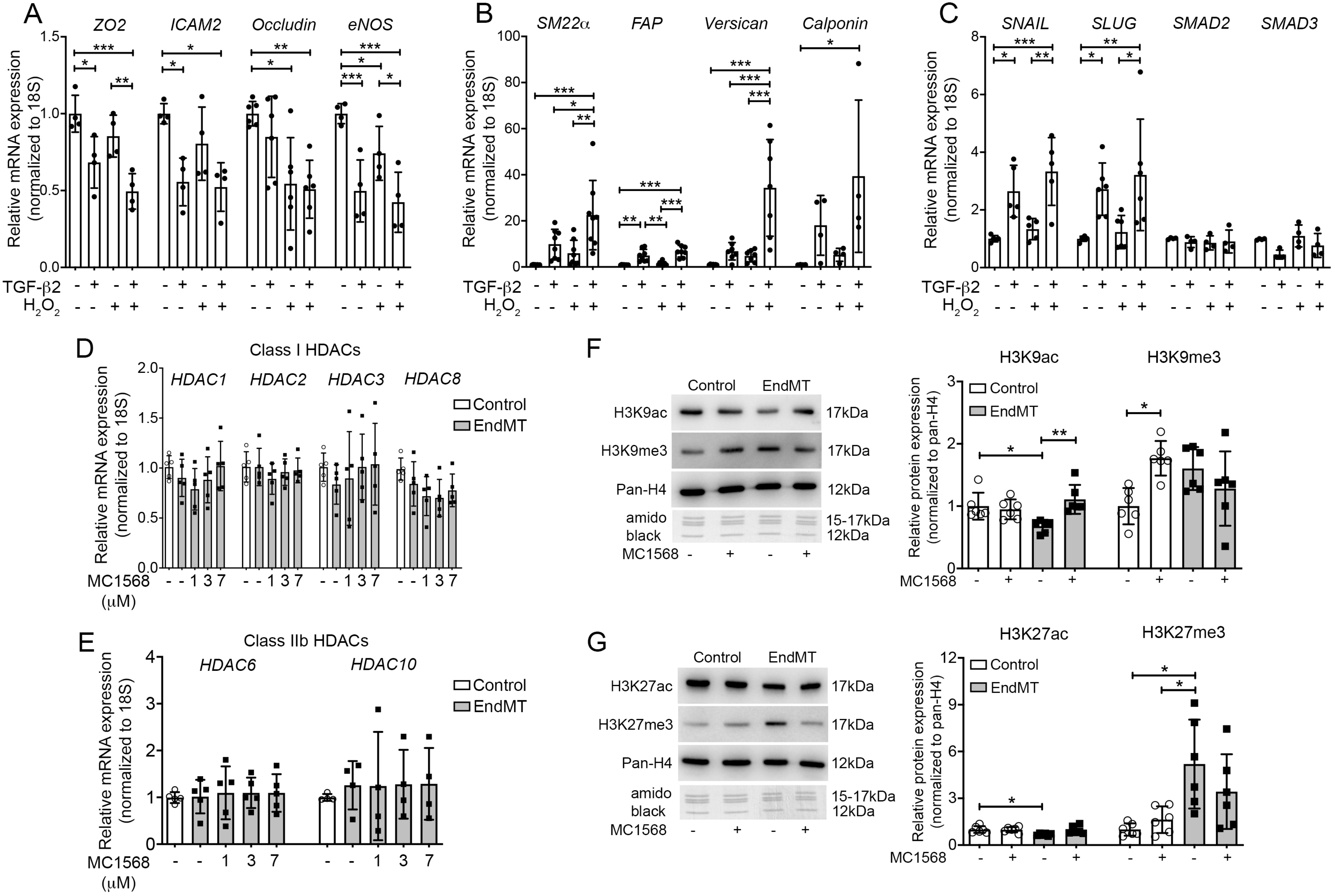

Supplemental Figure 2

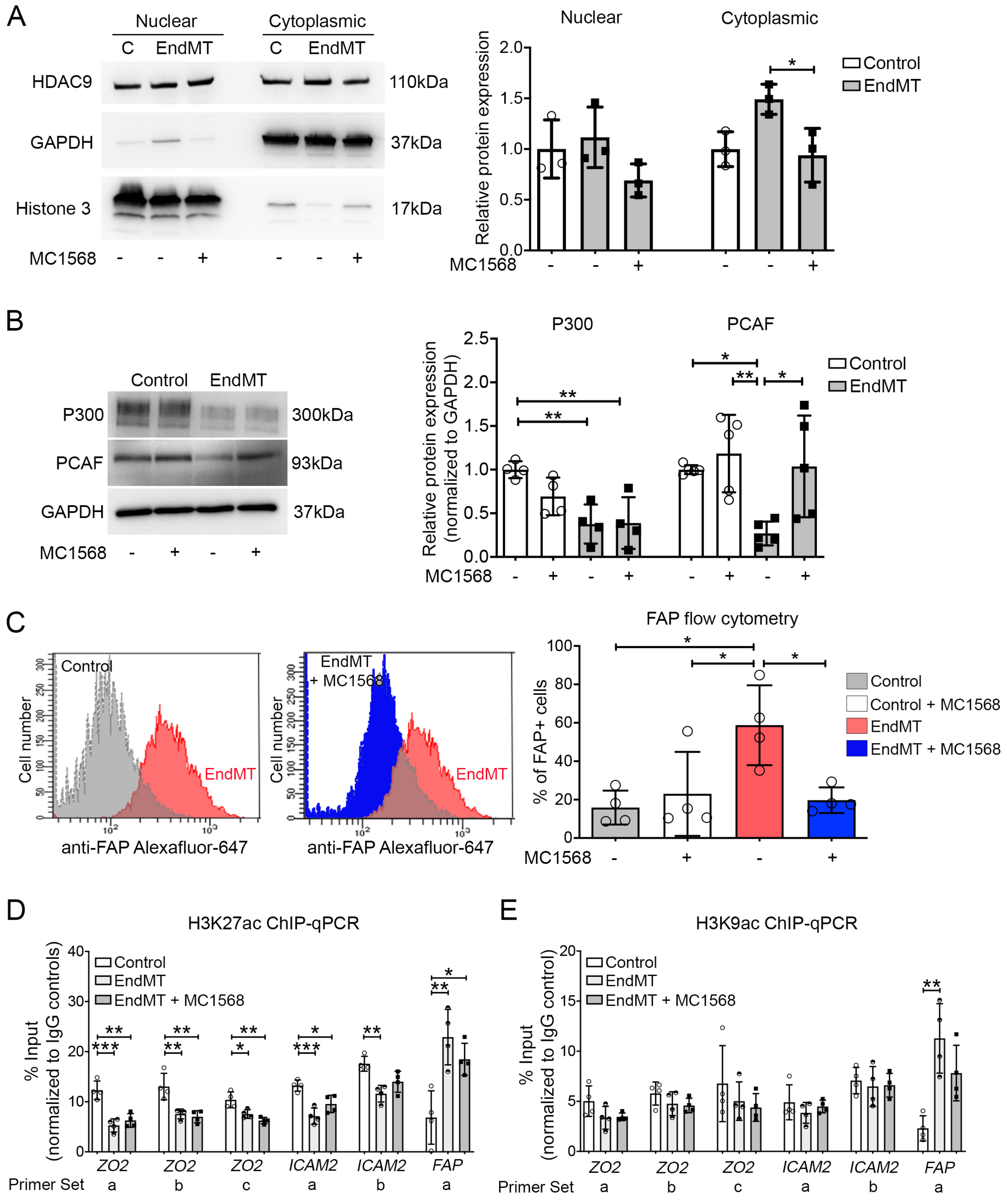

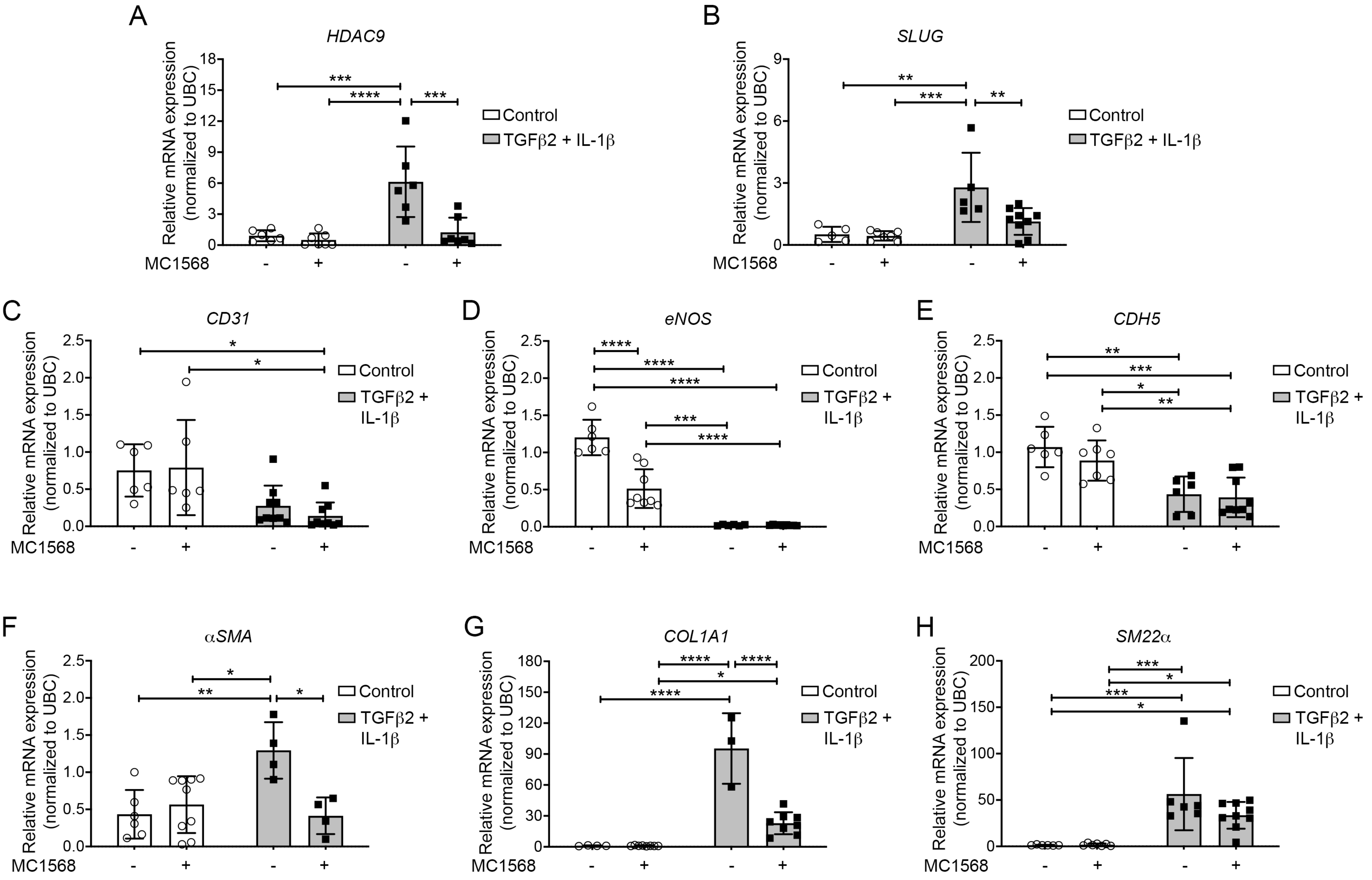
A
HDAC9
B
SLUG
C
CD31
D
eNOS
E
CDH5
F
αSMA
G
COL1A1
H
SM22α
Relative mRNA expression (normalized to UBC)
Control
TGFβ2 + IL-1β
MC1568
- + - +

Supplemental Figure 4

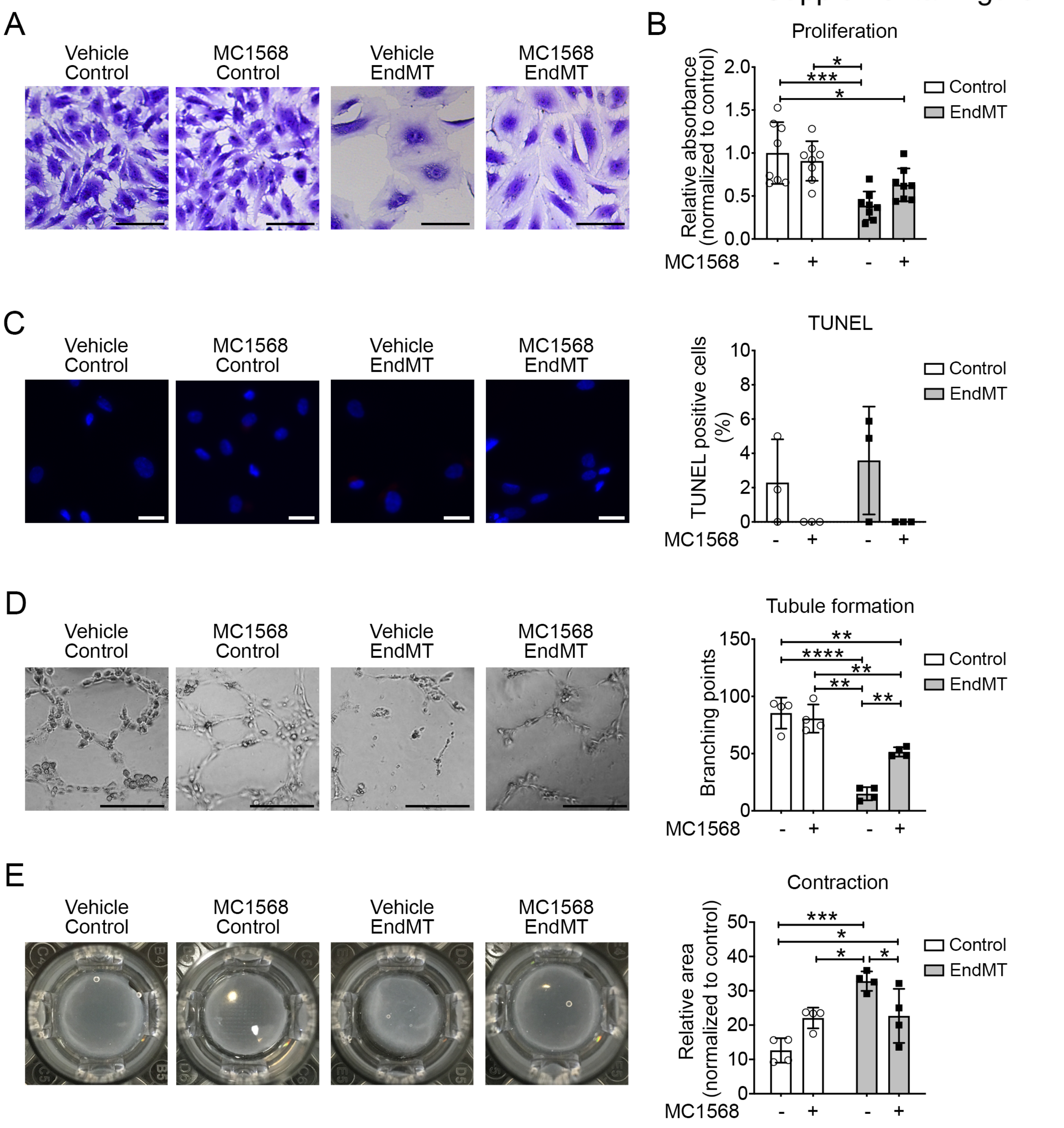

Supplemental Figure 5

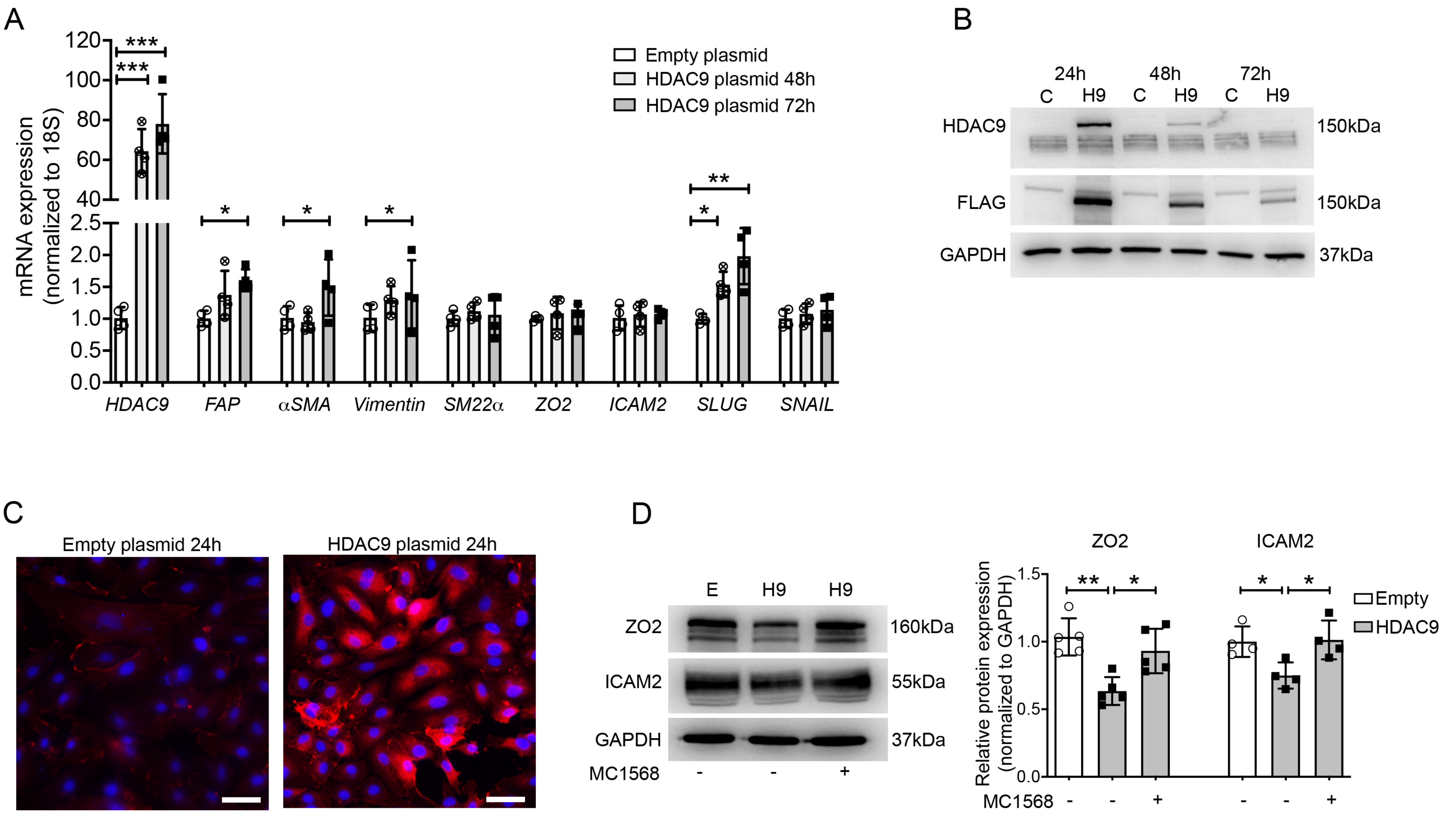

Supplemental Figure 6

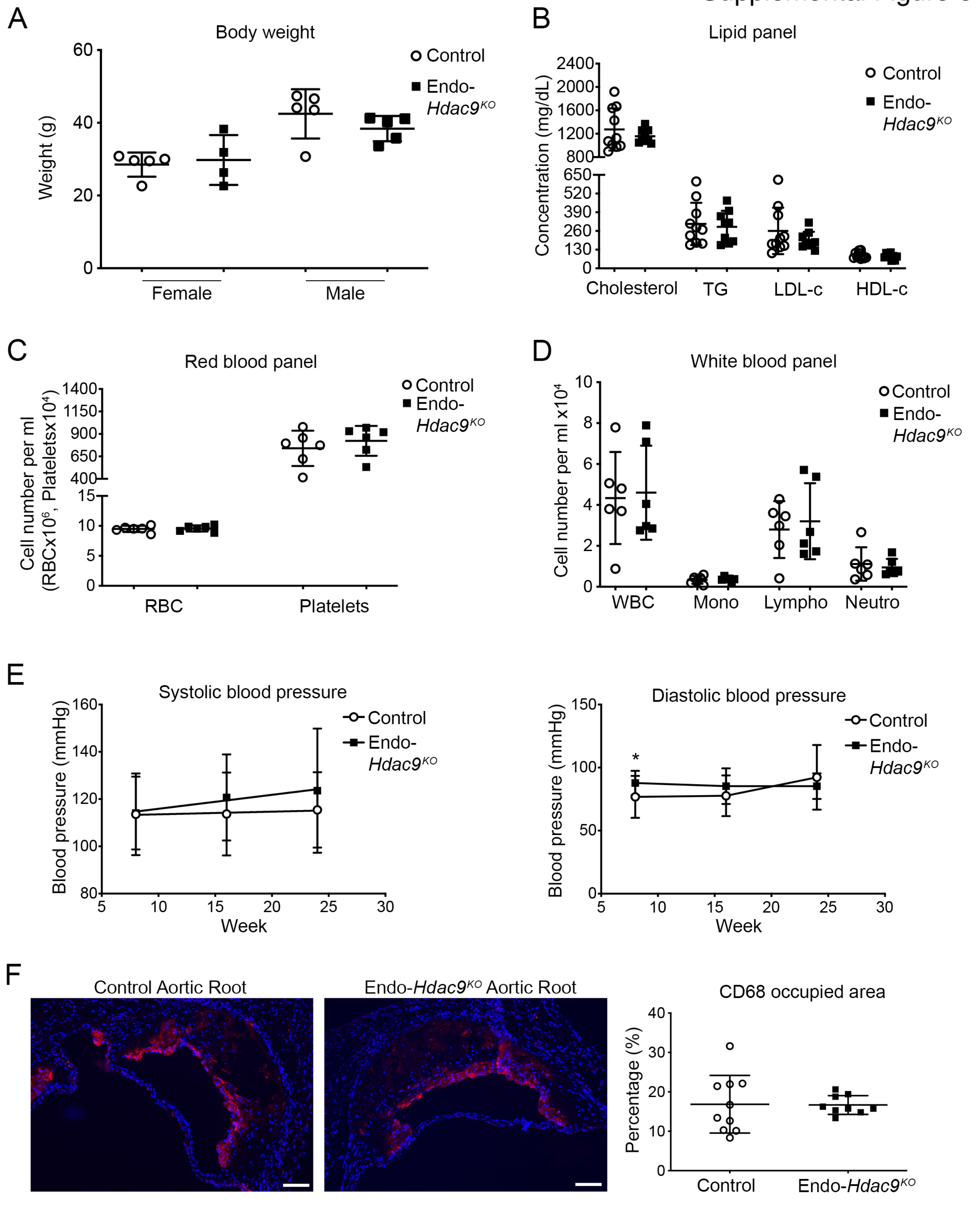

Supplemental Figure 7

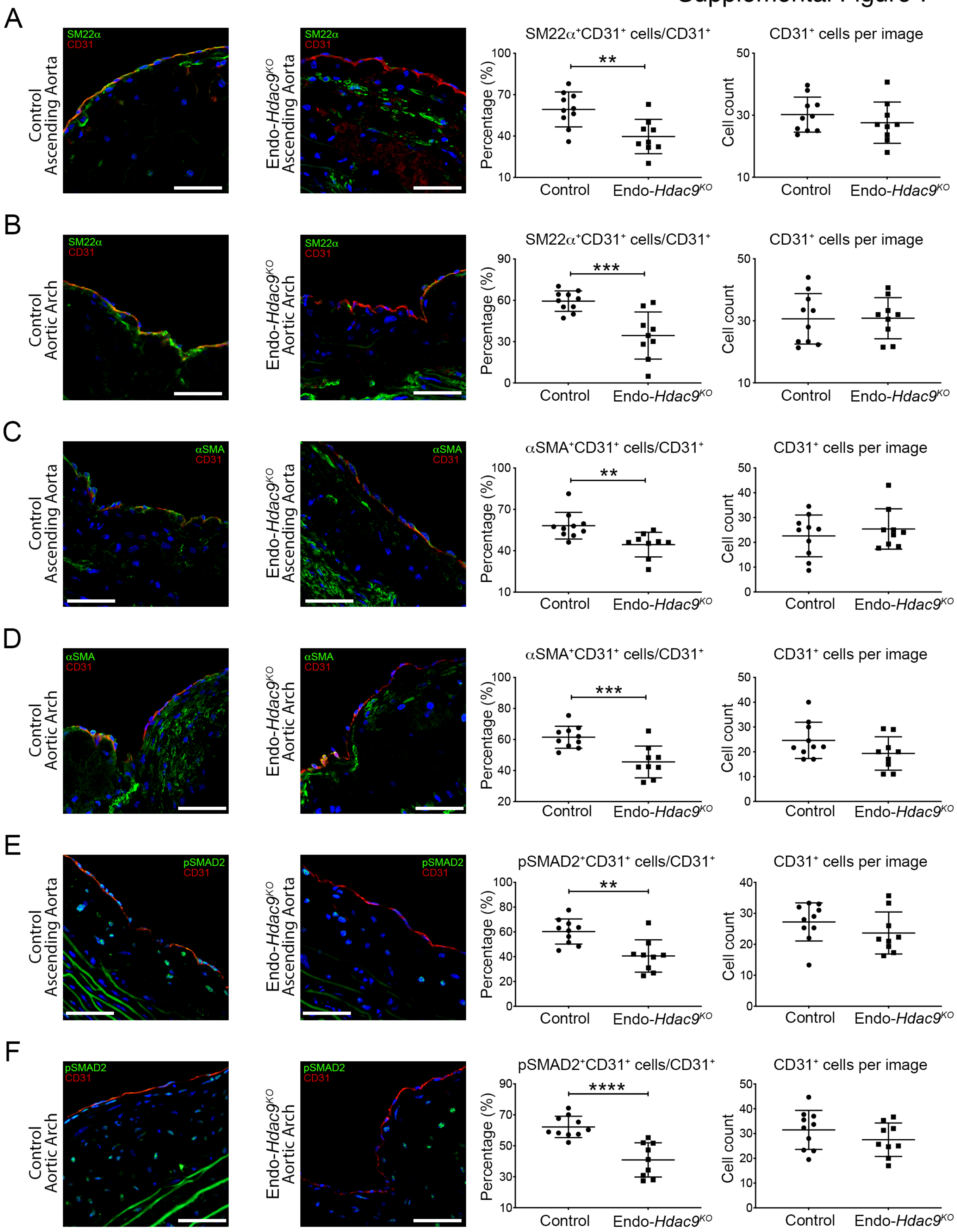

Supplemental Figure 8

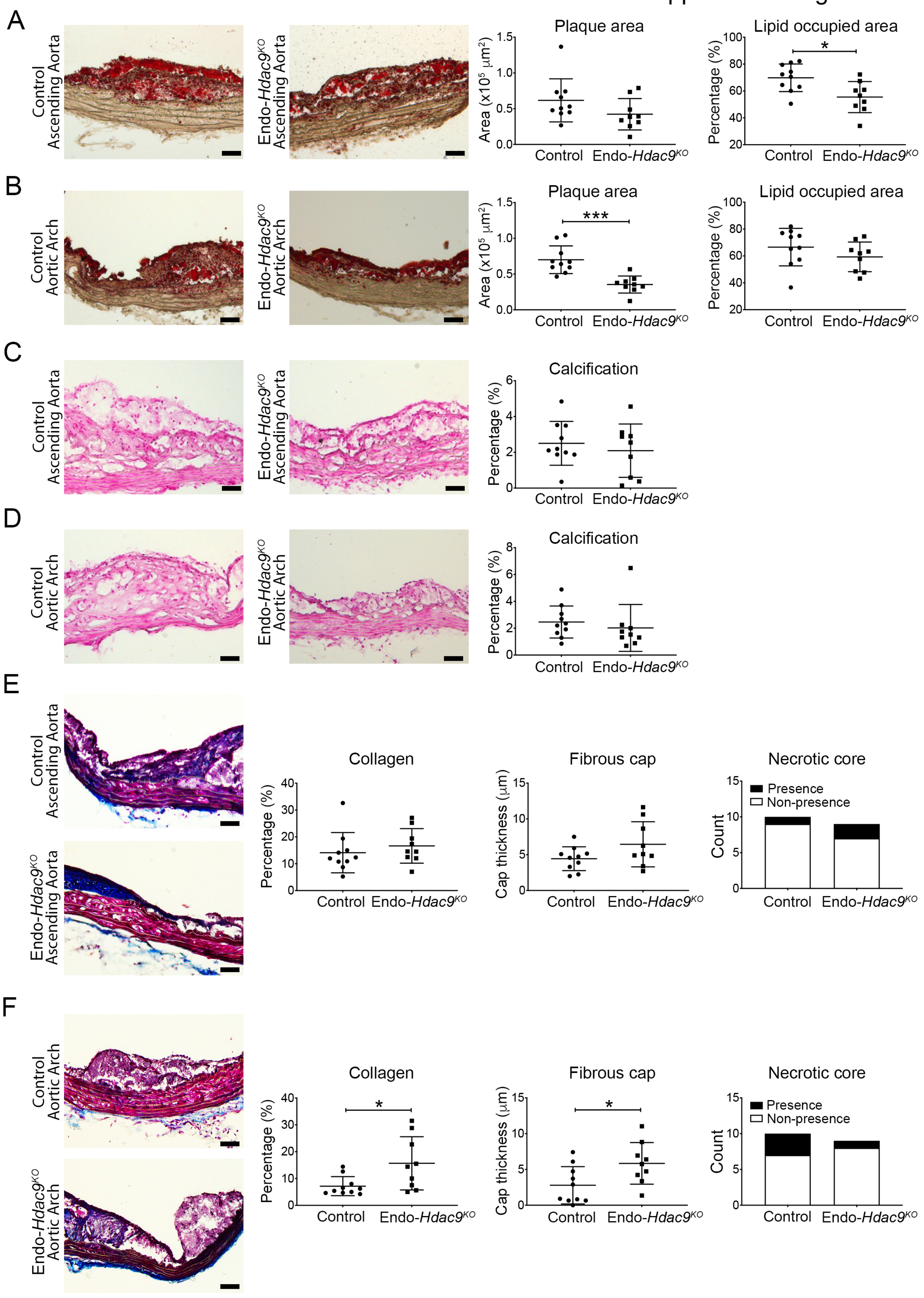

Supplemental Figure 9

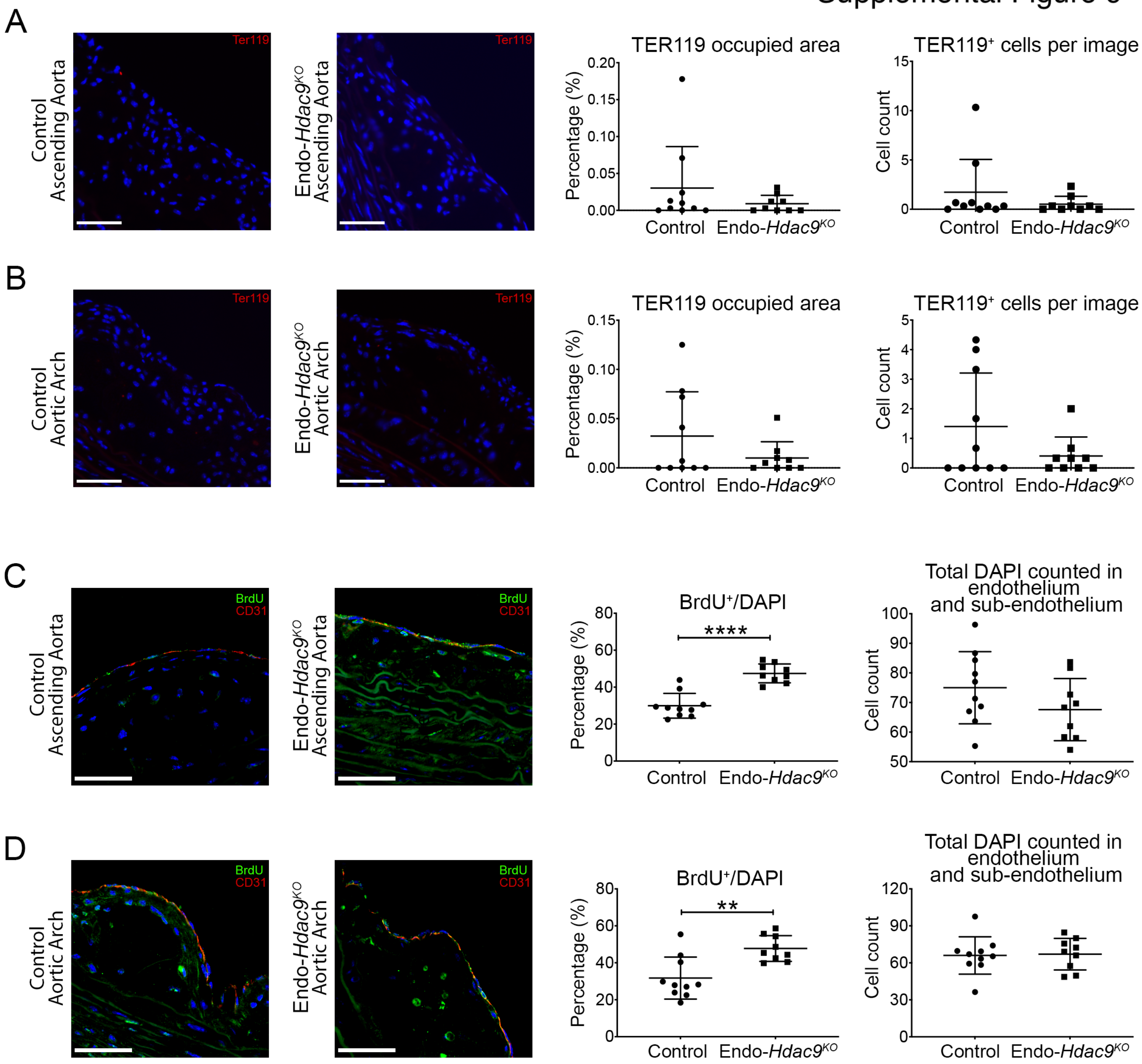

Supplemental Figure 10

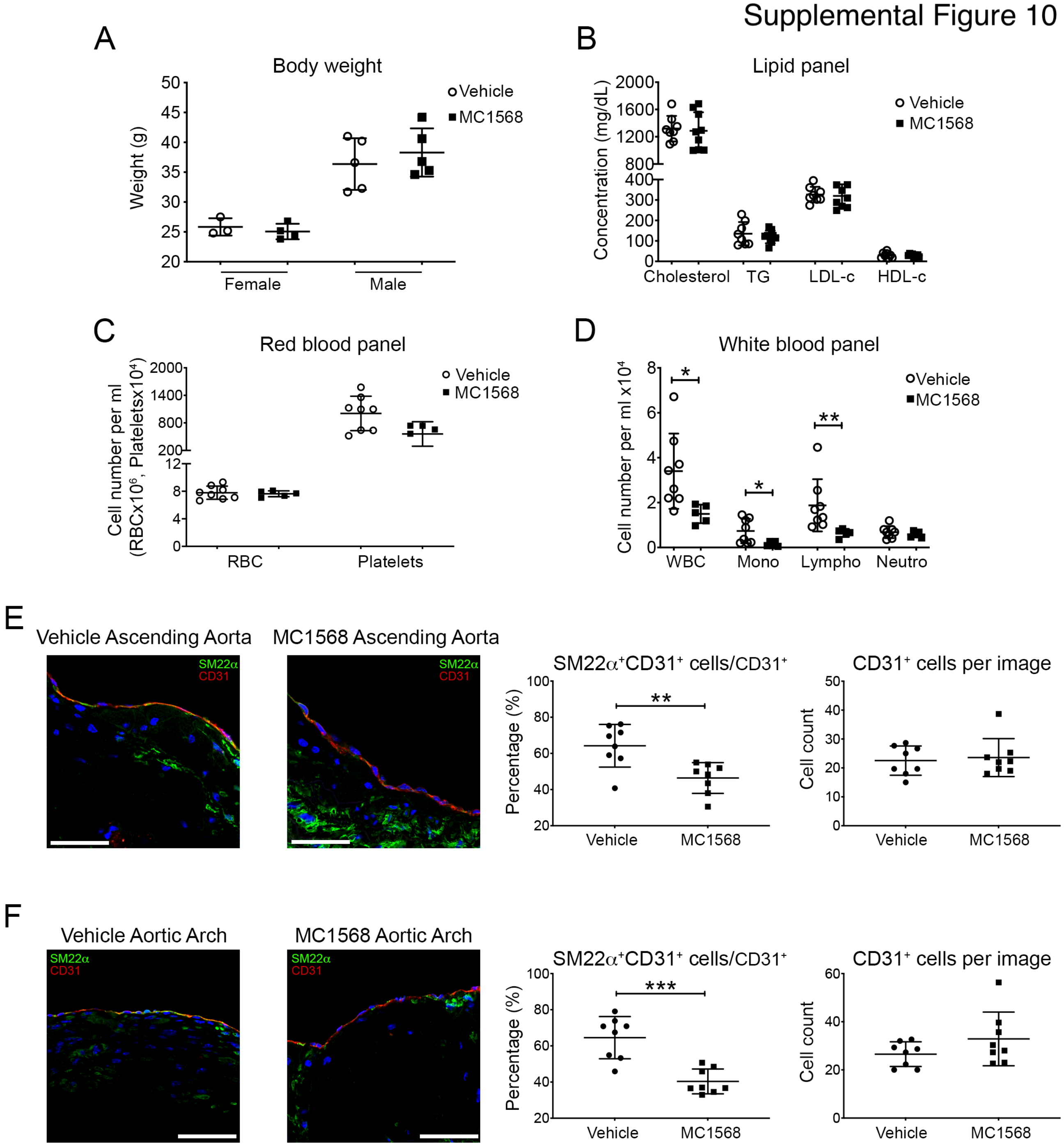

Supplemental Figure 11

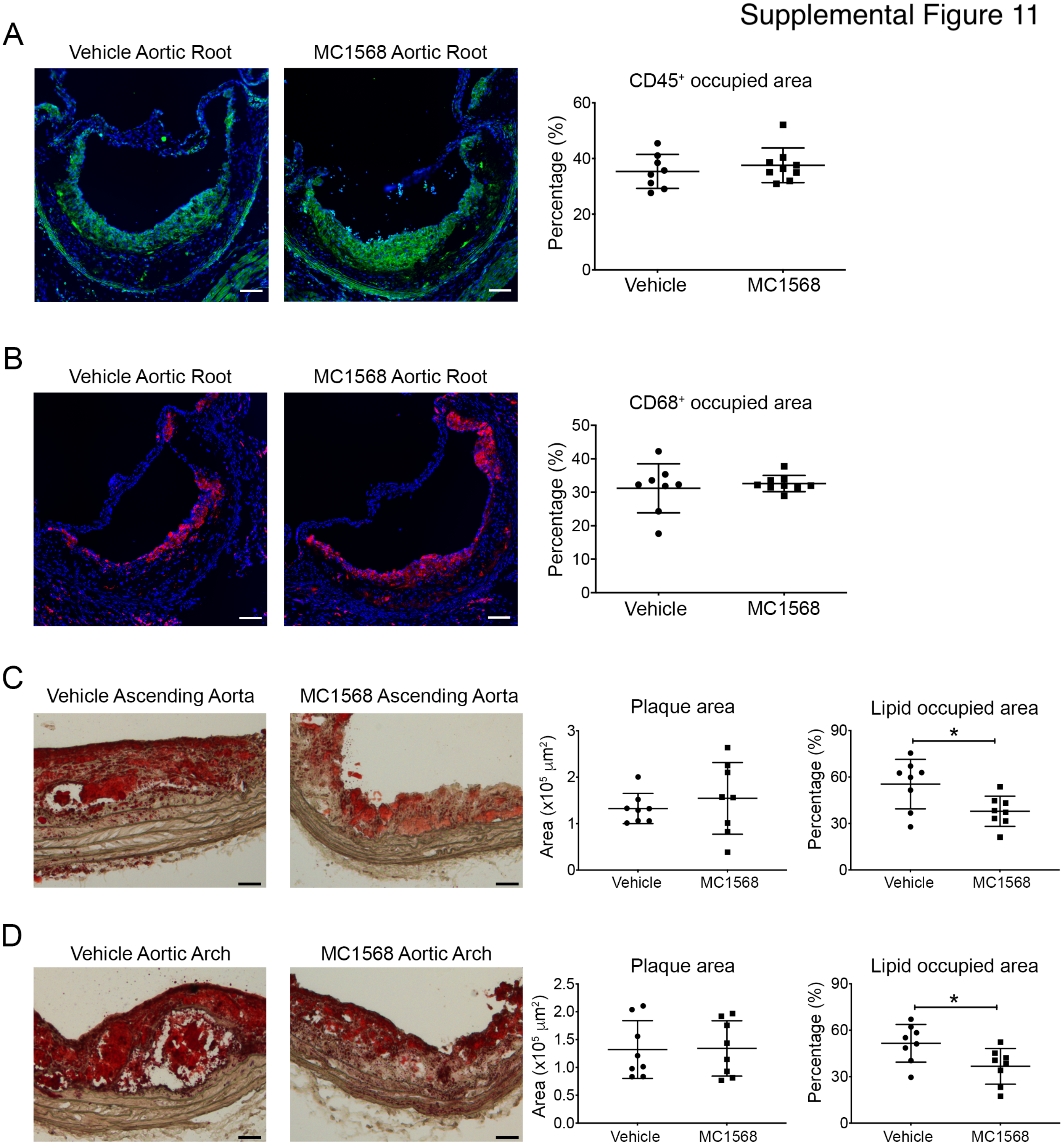

Full uncut gels

Figure 1

Figure 1A

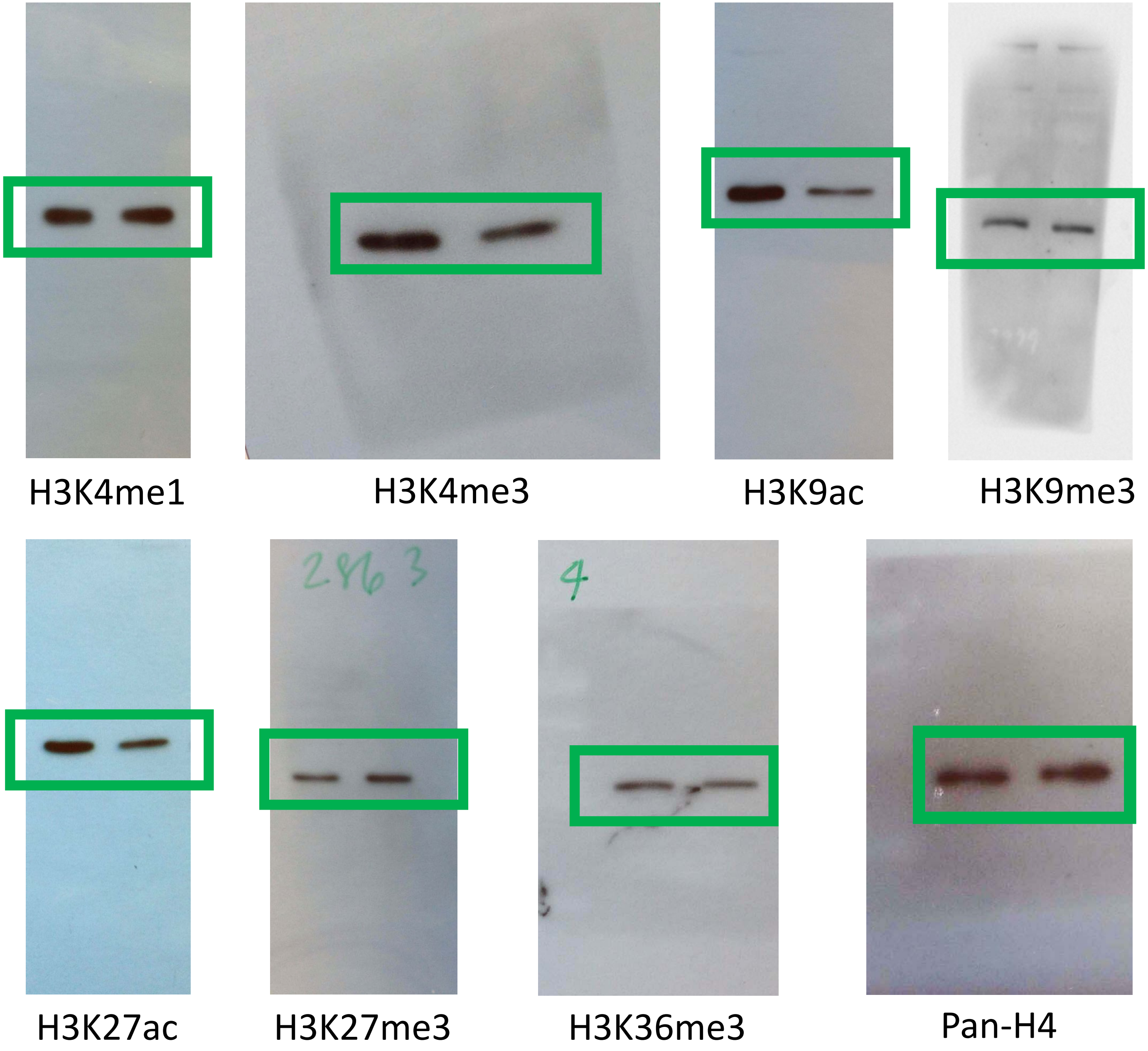

Figure 1

Figure 1E

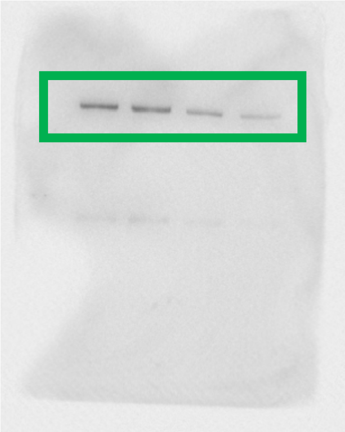

HDAC4

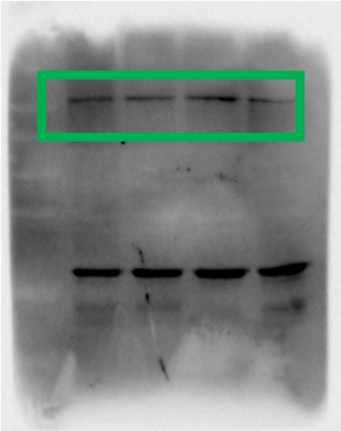

HDAC5

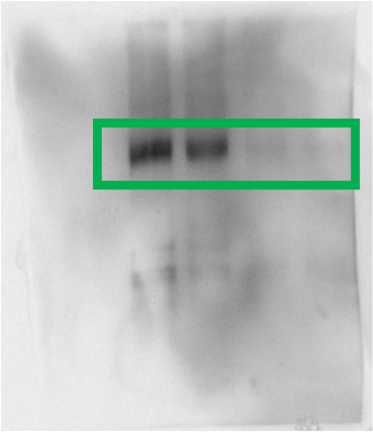

HDAC7

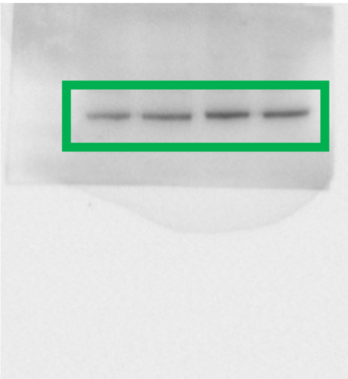

HDAC9

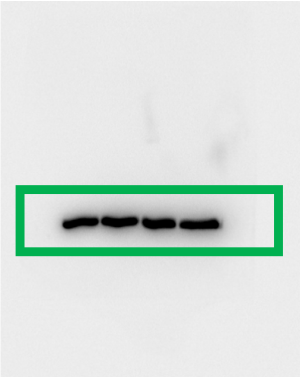

GAPDH

The lanes in green box correspond to those shown in the manuscript.

Figure 1

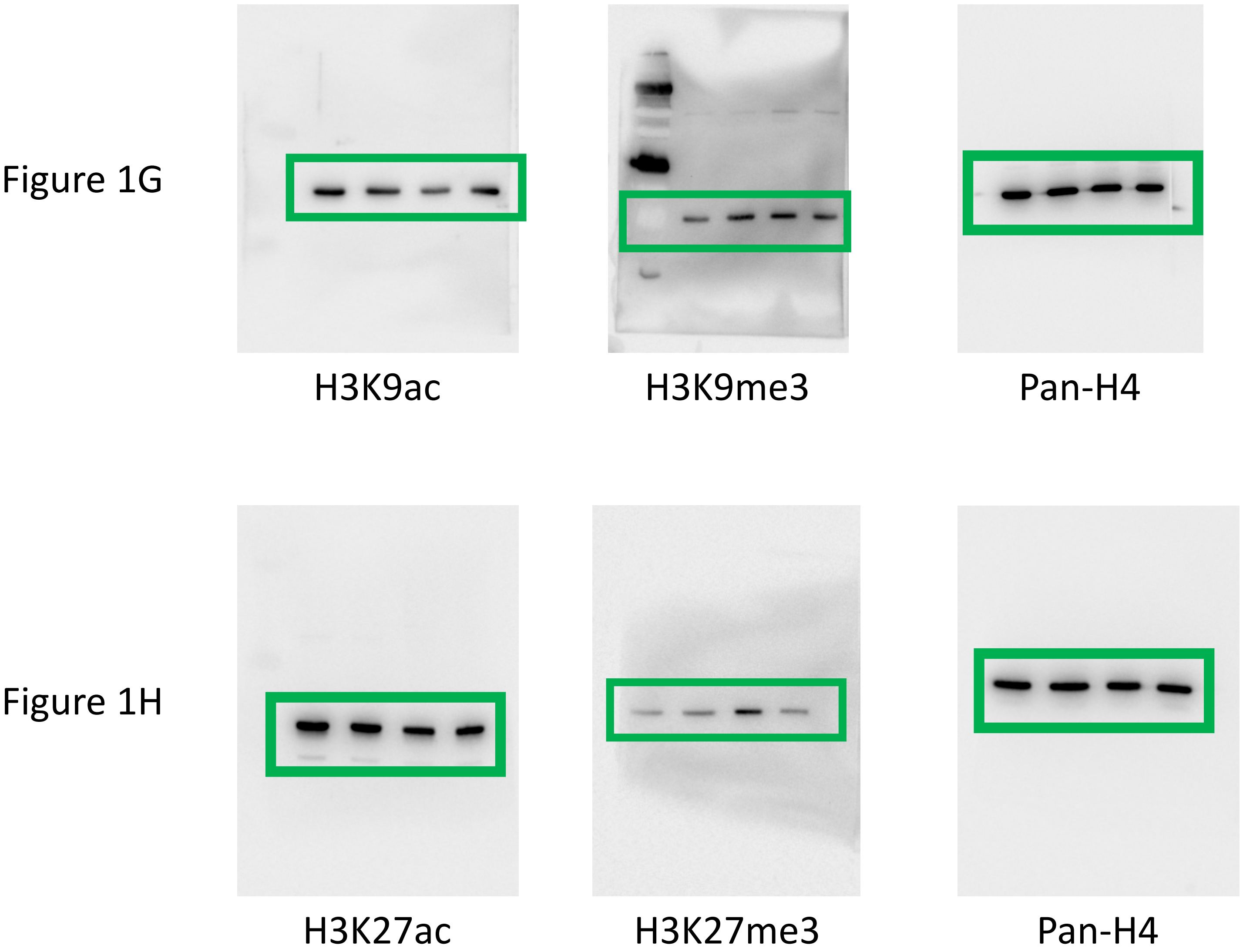

The lanes in green box correspond to those shown in the manuscript.

Figure 2

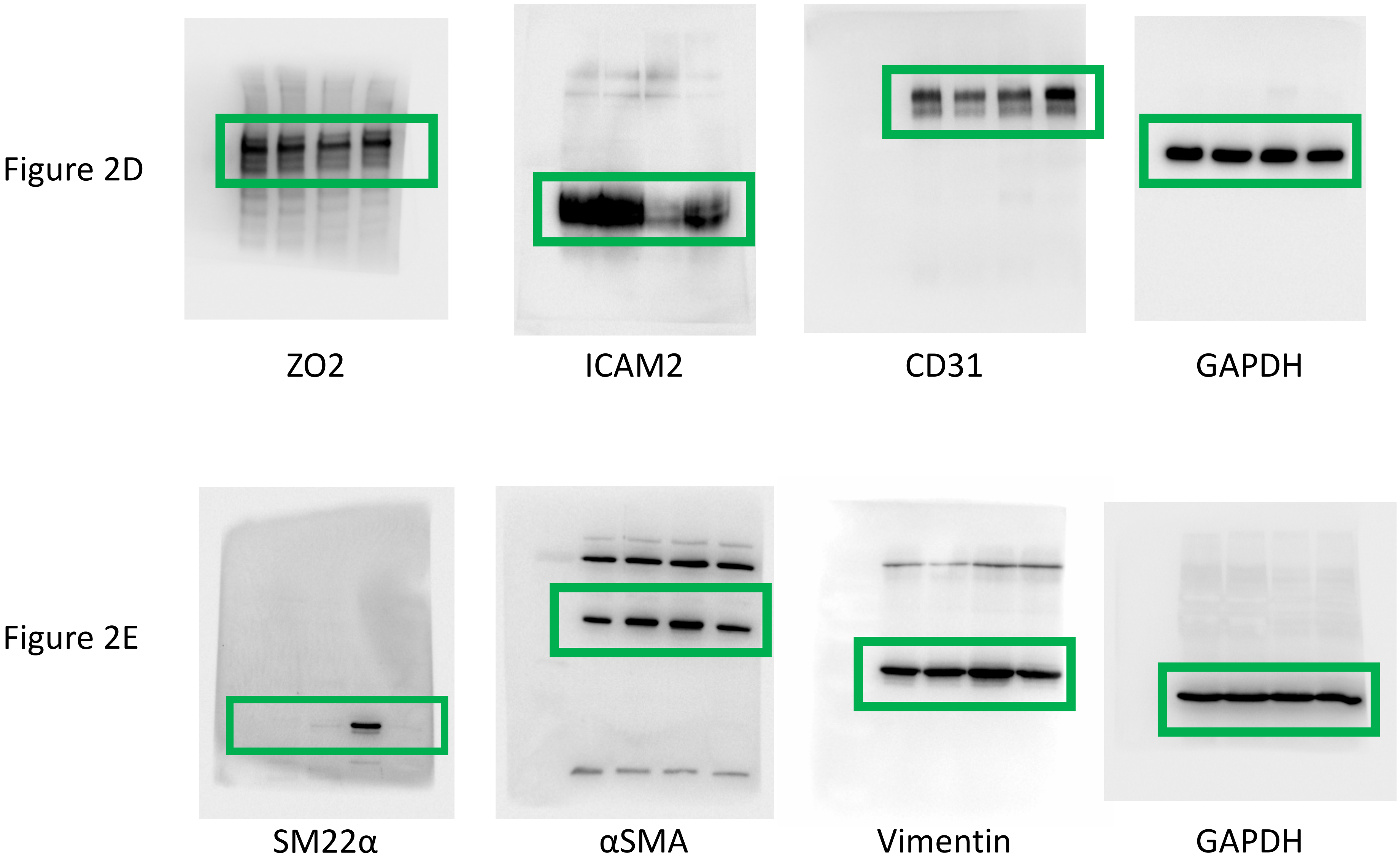

The lanes in green box correspond to those shown in the manuscript.

Figure 2

Figure 2F

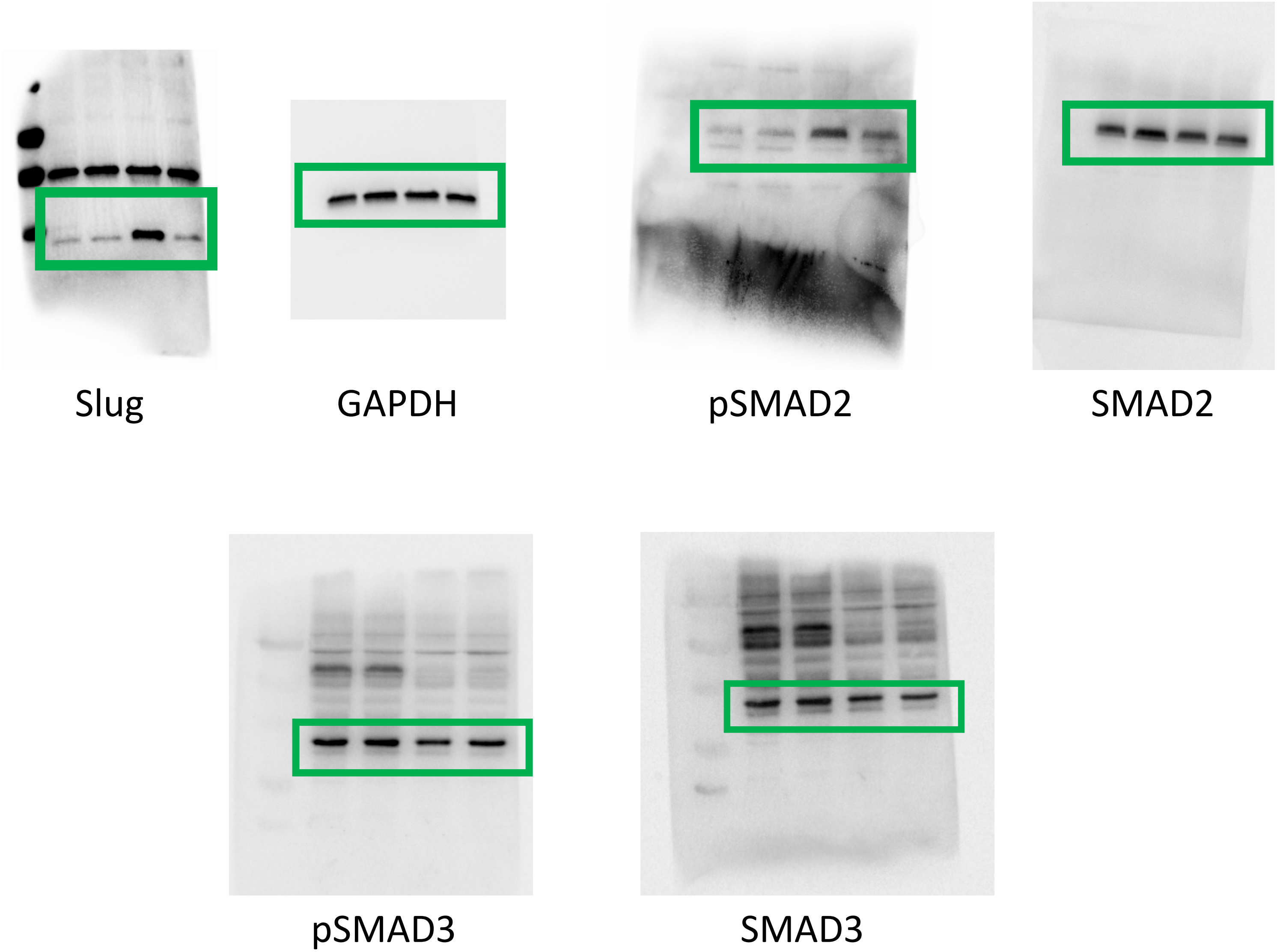

The lanes in green box correspond to those shown in the manuscript.

Supplemental Figure 1

Supplemental Figure 1F

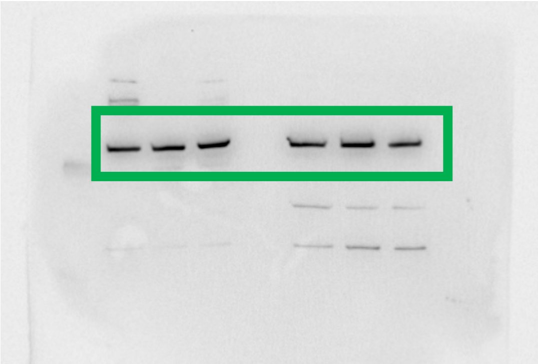

HDAC9

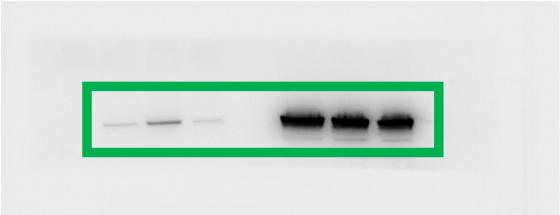

GAPDH

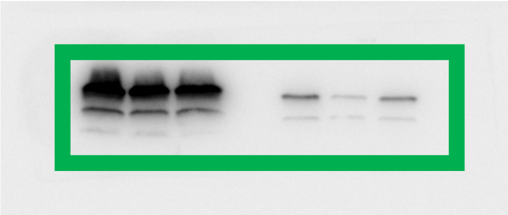

Histone 3

Supplemental Figure 1G

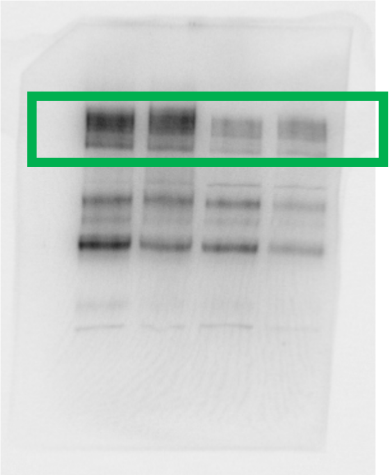

p300

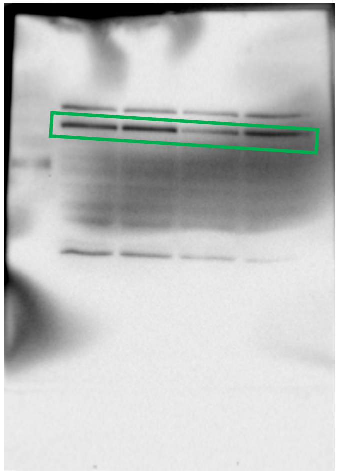

PCAF

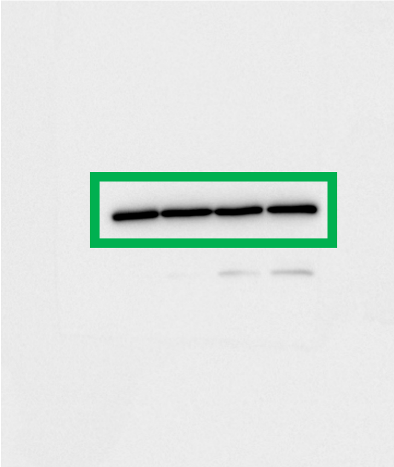

GAPDH

The lanes in green box correspond to those shown in the manuscript.

Supplemental Figure 5

Supplemental Figure 5B

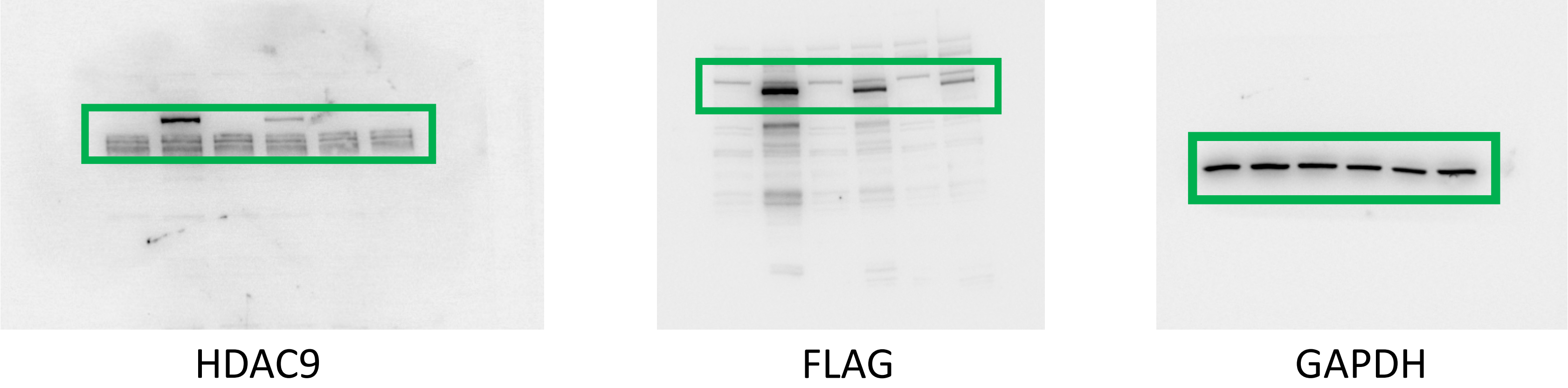

Supplemental Figure 5D

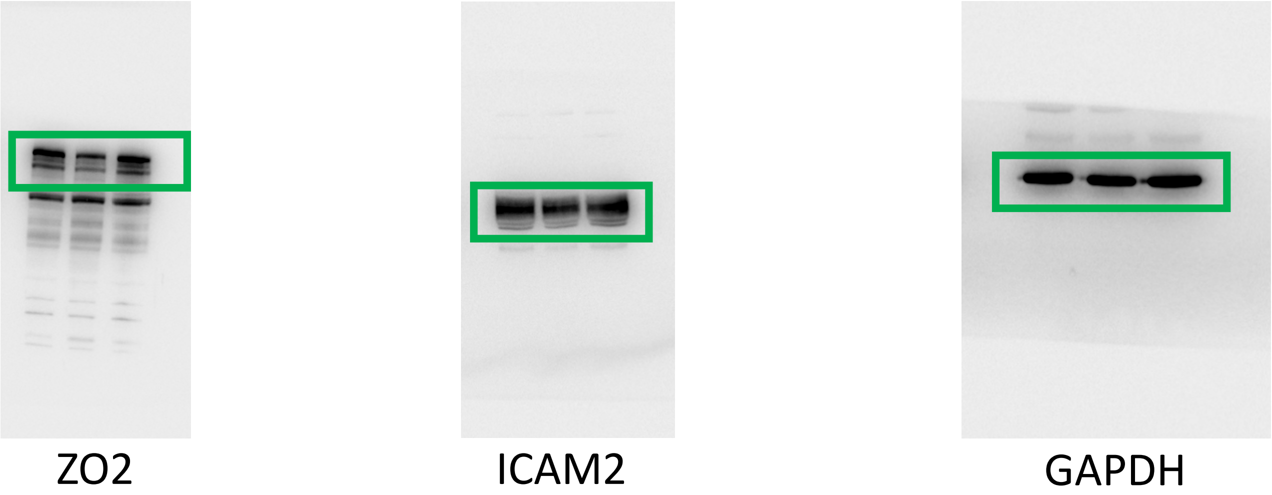

The lanes in green box correspond to those shown in the manuscript.
